# CD57-positive CD8 + T cells define the response to anti-programmed cell death protein-1 immunotherapy in patients with advanced non-small cell lung cancer

**DOI:** 10.1038/s41698-024-00513-0

**Published:** 2024-01-31

**Authors:** Wenjia Sun, Fengqi Qiu, Jing Zheng, Liangjie Fang, Jingjing Qu, Shumeng Zhang, Nan Jiang, Jianying Zhou, Xun Zeng, Jianya Zhou

**Affiliations:** 1 Department of Respiratory Disease, Thoracic Disease Center, The First Affiliated Hospital, College of Medicine, Zhejiang University, Hangzhou, China; 2 Cancer Center, Department of Pulmonary and Critical Care Medicine, Zhejiang Provincial People’s Hospital (Affiliated People’s Hospital), Hangzhou Medical College, Hangzhou, China; 3 State Key Laboratory for Diagnosis and Treatment of Infectious Diseases, National Clinical Research Center for Infectious Diseases, National Medical Center for Infectious Diseases, Collaborative Innovation Center for Diagnosis and Treatment of Infectious Diseases, The First Affiliated Hospital, Zhejiang University School of Medicine, Hangzhou, China

**Keywords:** Cancer immunotherapy, Non-small-cell lung cancer

## Abstract

Immune checkpoint inhibitors have transformed the treatment landscape of non-small cell lung cancer (NSCLC). However, accurately identifying patients who will benefit from immunotherapy remains a challenge. This study aimed to discover potential biomarkers for predicting immunotherapy response in NSCLC patients. Single-cell mass cytometry (CyTOF) was utilized to analyze immune cell subsets in peripheral blood mononuclear cells (PBMCs) obtained from NSCLC patients before and 12 weeks after single-agent immunotherapy. The CyTOF findings were subsequently validated using flow cytometry and multiplex immunohistochemistry/immunofluorescence in PBMCs and tumor tissues, respectively. RNA sequencing (RNA-seq) was performed to elucidate the underlying mechanisms. In the CyTOF cohort (*n* = 20), a high frequency of CD57^+^CD8^+^ T cells in PBMCs was associated with durable clinical benefit from immunotherapy in NSCLC patients (*p* = 0.034). This association was further confirmed in an independent cohort using flow cytometry (*n* = 27; *p* < 0.001), with a determined cutoff value of 12.85%. The cutoff value was subsequently validated in another independent cohort (AUC = 0.733). We also confirmed the CyTOF findings in pre-treatment formalin-fixed and paraffin-embedded tissues (*n* = 90; *p* < 0.001). RNA-seq analysis revealed 475 differentially expressed genes (DEGs) between CD57^+^CD8^+^ T cells and CD57^-^CD8^+^ T cells, with functional analysis identifying DEGs significantly enriched in immune-related signaling pathways. This study highlights CD57^+^CD8^+^ T cells as a promising biomarker for predicting immunotherapy success in NSCLC patients.

## Introduction

With the widespread use of immune checkpoint inhibitors (ICIs), immunotherapy has shown epoch-making effects in non-small cell lung cancer (NSCLC), especially in terms of long-term survival^1^. Nevertheless, immunotherapy has only demonstrated long-term antitumor efficacy in a few patients with NSCLC^2,3^. Such low objective response rates may be due to tumor heterogeneity in systemic immunity, pathogenesis, histopathology, and the molecular basis of NSCLC^4–7^. Currently, programmed death-ligand 1 (PD-L1) expression is an acknowledged biomarker for predicting immunological efficacy in NSCLC and guiding the clinical practice of immunotherapy^8^, however, it is not a perfect biomarker for immunotherapeutic prediction in NSCLC because the use of this predictor is hampered by the overlap between responders and non-responders^9–12^. To date, significant efforts have been made to identify reliable biomarkers to predict immunotherapeutic efficacy in patients with NSCLC; however, robust biomarkers have not yet been established to drive clinical practice^13–16^. Thus, potential biomarkers to precisely identify patients who will benefit from immunotherapy before treatment initiation are urgently required.

Analysis of tumor samples is currently considered the standard method for identifying and characterizing immunotherapy biomarkers. Previous studies using high-dimensional single-cell analysis have revealed the composition of tumor microenvironment in NSCLC^17–21^. These findings were important for comprehending the functions of specific immune cell subsets, such as CD39^+^CD8^+^ T cells, in response to ICIs in NSCLC^21^. However, tumor biopsy is challenging because the procedure is invasive and may lead to inadequate sample collection. Peripheral blood, as a feasible and sensitive alternative, is appealing for investigating predictive biomarkers for immunotherapy because of its noninvasiveness, easy accessibility, and reproducibility in obtaining blood versus tissue samples^22^. Blood is also more homogeneous than tissues, making blood sampling easier and more consistent. Understanding the differential responses to immunotherapy necessitates knowledge of potential immune cell subsets and functions. In this regard, conventional fluorescent flow cytometry is inadequate for describing diverse tumor subpopulations because of the limited number of features that can be simultaneously analyzed. Therefore, to overcome this shortcoming, single-cell mass cytometry (CyTOF), which allows the measurement of up to 50 features in a single cell^23^, has been used for tumor-related research on peripheral blood. Currently, CyTOF has been used to explore the association between diverse cell subpopulations and clinical responses; for instance, it has been examining single-cell-based immune biomarkers for predicting immunotherapy efficacy in advanced melanoma^24^.

Our study aimed to investigate the immune signatures in peripheral blood associated with responsiveness to programmed cell death protein-1 (PD-1) inhibitor and identify a responsiveness-associated predictive signature. We examined immune cell subpopulations in the peripheral blood of patients with advanced NSCLC before and during PD-1 inhibitor monotherapy using high-dimensional single-cell analysis via CyTOF. Indeed, we discovered that the baseline frequency of CD57^+^CD8^+^ T cells could help identify patients with durable clinical benefit (DCB) and no durable clinical benefit (NDB) to ICIs before therapy. An independent validation cohort of patients with NSCLC revealed that the frequency of baseline CD57^+^CD8^+^ T cells was higher in DCB group and had high sensitivity and specificity for predicting the responsiveness to ICIs, with a determined cutoff value of 12.85%. The cutoff value was subsequently validated in another independent cohort. We confirmed that the frequency of CD57^+^CD8^+^ T cells in tumor tissues was associated with responsiveness using multiplex immunohistochemistry / immunofluorescence (mIHC/IF). Transcriptome analysis of pre-treatment blood revealed 475 differentially expressed genes (DEGs) between CD57^+^CD8^+^ T and CD57^-^CD8^+^ T cells, and the DEGs were significantly enriched in immune-related signaling pathways. This provides a novel and strong predictive biomarker that can be used for the effective response assessment of ICIs before therapy in patients with NSCLC.

## Results

### Major peripheral immune compositions were essentially the same among patients with NSCLC with distinct responses to anti-PD-1 immunotherapy

**Fig. 1:**
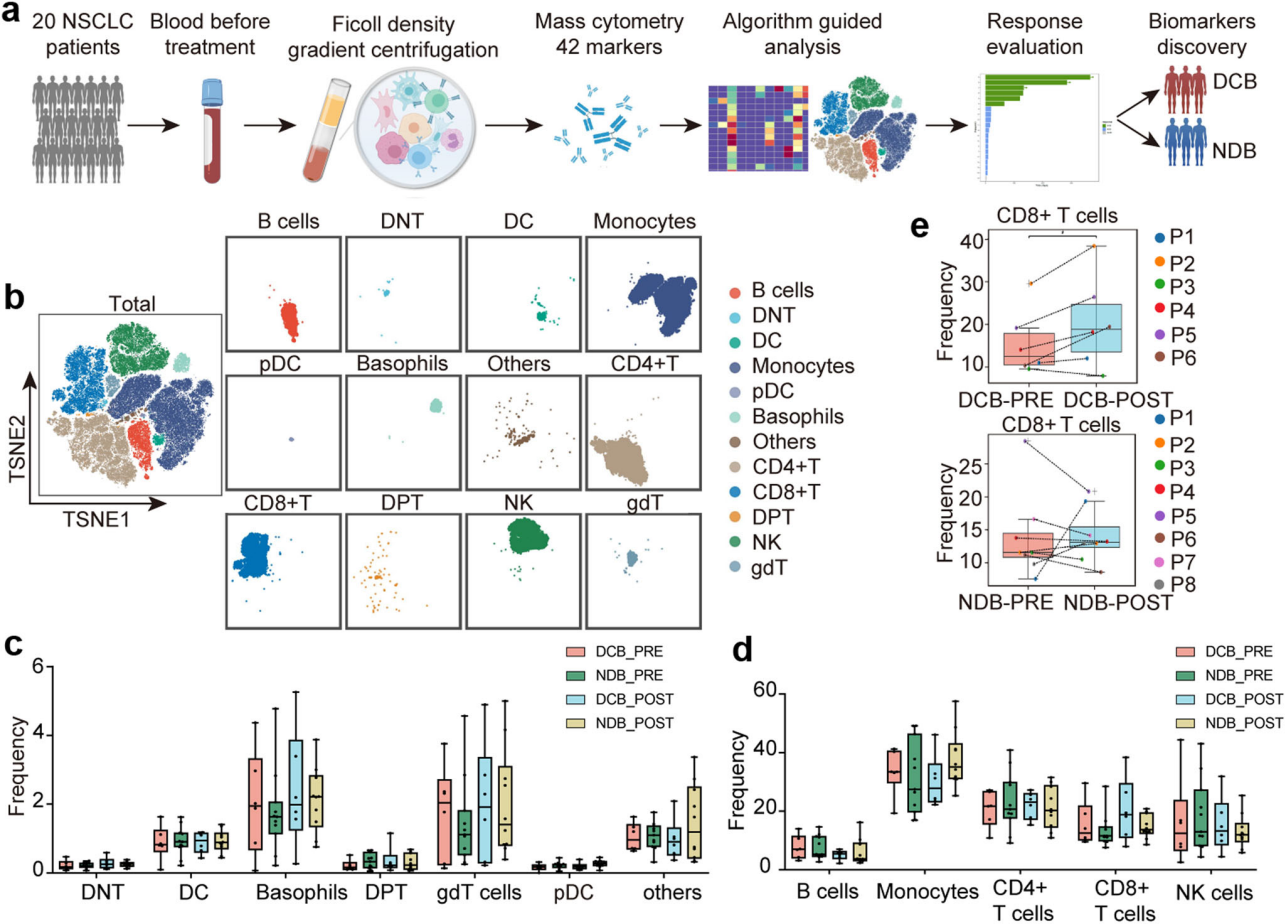
Major immune lineages of PBMCs from patients with NSCLC revealed by CyTOF. **a** Experimental design and analysis flow for CyTOF. **b** t-SNE plot identifying the 12 major immune cell subsets from PBMCs, including CD4^+^ T cells, CD8^+^ T cells, γδT, DNT, DPT, monocytes, DC, pDC, B cells, NK cells, basophils and other cells in all samples, colored by major immune cell subsets. **c**, **d** Boxplots demonstrating the frequencies of the 12 immune cell subsets in CD66b^−^ cells among DCB and NDB patients before and after immunotherapy. **e** Paired PBMC samples analysis before and after immunotherapy demonstrating the changes in frequencies of the CD8^+^ T cell subset among DCB and NDB patients. *p < 0.05. PBMCs peripheral blood mononuclear cells; NSCLC non-small cell lung cancer, CyTOF cytometry by time of flight, t-SNE t-distributed Stochastic Neighbor Embedding, DNT double-negative T cells, DPT double-positive T cells, DC dendritic cells, pDC plasmacytoid dendritic cells, NK cells natural killer cells, DCB durable clinical benefit, NDB no durable clinical benefit.

CyTOF analysis was performed on 34 peripheral blood mononuclear cells (PBMCs) samples from a prospective discovery cohort comprising 20 patients with NSCLC treated with PD-1 inhibitor monotherapy to perform an in-depth evaluation of the immunological profiles of PBMCs in NSCLC (Fig. 1a). A predefined 42-marker panel was specifically designed for patients with NSCLC, including phenotypic and functional markers, to define the composition and function of leukocytes (Supplementary Table 1). According to the clinical efficacy after immunotherapy, one patient was excluded (death due to severe adverse events), leaving 19 patients for the final analysis (6 and 13 in the DCB and NDB groups, respectively). Detailed clinical information was presented in Table 1. Samples were collected from these 19 patients at different time points, including before treatment (*n* = 17) and 12 weeks after treatment (*n* = 16). Of these, 14 (DCB, *n* = 6; NDB, *n* = 8) included paired blood samples (Supplementary Table 2).

**Table 1:** The clinical characteristics of advanced NSCLC in CyTOF cohort (*n* (%)).

Characteristics	CyTOF cohort (*n* = 19)	DCB (*n* = 6)	NDB (*n* = 13)	*p* valvue
Gender				0.554
Male (M)	17 (89.5)	5 (83.3)	12 (92.3)	
Female (F)	2 (10.5)	1 (16.7)	1 (7.7)	
Age (years)				0.216
<65	7 (36.8)	1 (16.7)	6 (46.2)	
≥65	12 (63.2)	5 (83.3)	7 (53.8)	
Smoking				0.372
YES	15 (78.9)	4 (66.7)	11 (84.6)	
NO	4 (21.1)	2 (33.3)	2 (15.4)	
Line of therapy				0.636
First	8 (42.1)	3 (50.0)	5 (38.5)	
Second	11 (57.9)	3 (50.0)	8 (61.5)	
Histological type				0.111
LUAD	5 (26.3)	3 (50.0)	2 (15.4)	
LUSC	14 (73.7)	3 (50.0)	11 (84.6)	
Tumor invasion (T_stage)				0.636
≤3	8 (42.1)	3 (50.0)	5 (38.5)	
4	11 (57.9)	3 (50.0)	8 (61.5)	
Lymph node (N_stage)				0.750
≤2	15 (78.9)	5 (83.3)	10 (76.9)	
3	4 (21.1)	1 (16.7)	3 (23.1)	
Metastasis (M_stage)				0.750
M0	4 (21.1)	1 (16.7)	3 (23.1)	
M1	15 (78.9)	5 (83.3)	10 (76.9)	
Tumor stage				0.750
Stage IIIB/C	4 (21.1)	1 (16.7)	3 (23.1)	
Stage IV	15 (78.9)	5 (83.3)	10 (76.9)	

*NSCLC* non-small cell lung cancer, *CyTOF* cytometry by time of flight, *DCB* durable clinical benefit, *NDB* no durable clinical benefit, *LUAD* lung adenocarcinoma, *LUSC* lung squamous cell carcinoma.

To analyze immune cells without granulocytes, we clustered CD66b^−^ cells, and characterized 12 major clusters according to the main immune cell markers, including CD4^+^ T cells, CD8^+^ T cells, double-negative T cells (DNT), double-positive T cells (DPT), γδT, monocytes, dendritic cells (DC), plasmacytoid dendritic cells (pDC), B cells, natural killer cells (NK), basophils and other cells among patients with DCB and NDB before and after immunotherapy, as displayed using t-distributed Stochastic Neighbor Embedding (t-SNE) analysis (Fig. 1b, Supplementary Fig. 1a, b). Signature markers (e.g., CD3, CD19, and CD56) revealed the distribution of immune clusters (T, B, and NK cells; Supplementary Fig. 1c). We compared the frequencies of immune cell subsets between the two groups before and after immunotherapy; however, no significant differences were observed (Fig. 1c, d). Next, we performed a paired analysis of samples from 14 patients with paired samples (DCB, *n* = 6; NDB, *n* = 8) before and after immunotherapy. In the DCB group, the frequency of CD8^+^ T cells significantly increased after anti-PD-1 immunotherapy compared to that before immunotherapy (paired *t*-test, *p* = 0.045; Fig. 1e); however, no difference was found in the NDB group (paired *t*-test, *p* > 0.05; Fig. 1e, Supplementary Fig. 2).

### The frequency of the CD57^+^CD8^+^ T cell subset was higher in the DCB group than in the NDB group

**Fig. 2:**
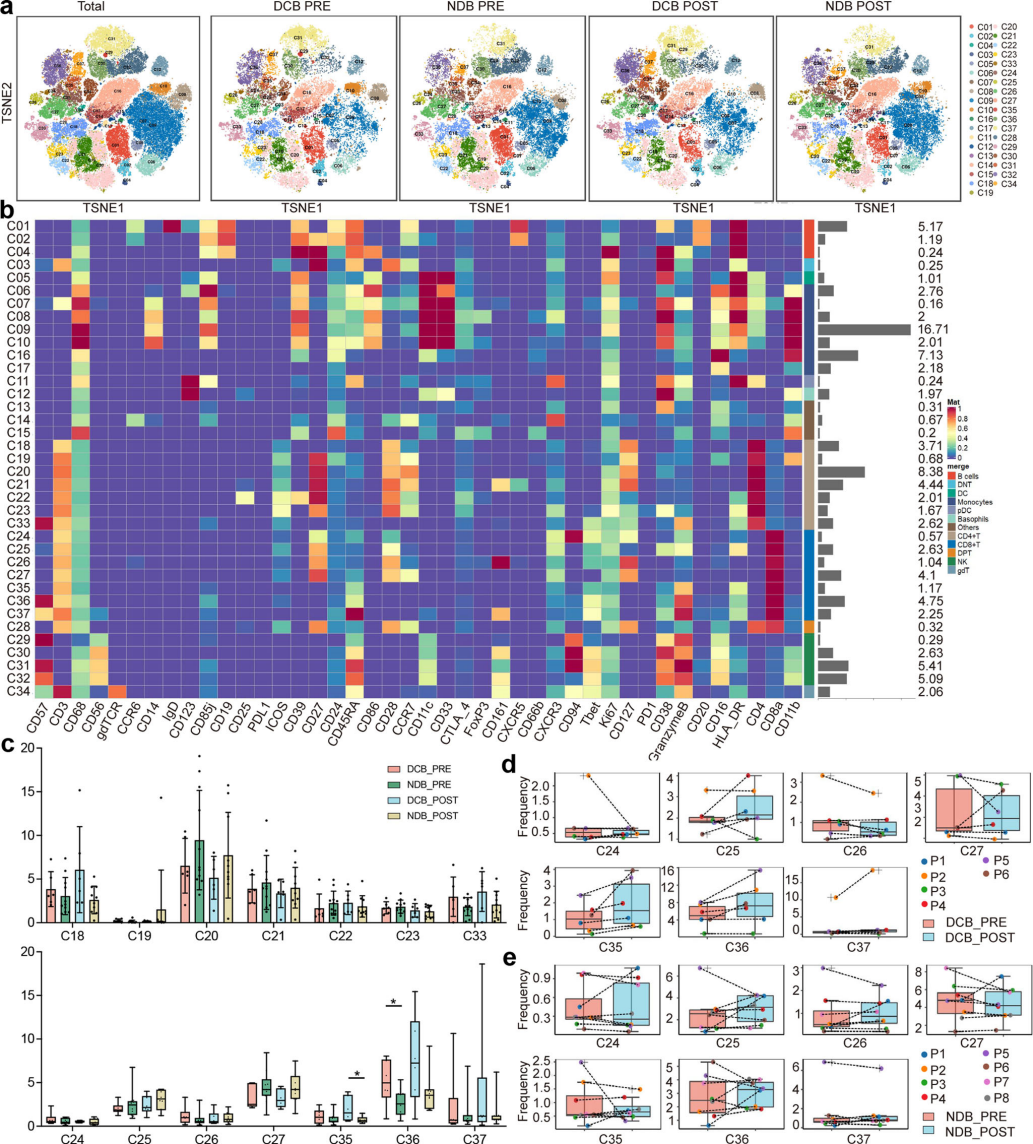
Identification of the peripheral immune cell populations in patients with NSCLC. **a** t-SNE plot identifying 37 immune cell clusters from PBMCs in all patients, and in DCB and NDB patients before and after immunotherapy, colored by immune cell subsets. **b** Heatmap showing the normalized mean expression of 42 membranous or intracellular markers to identify the phenotypes of the 12 major immune cell clusters. We characterized one cluster in DC, pDC, Basophils, DNT, DPT and γδT, three clusters in B cells, four clusters in NK cells, seven clusters in monocytes, seven CD4^+^ T cells, seven CD8^+^ T cells, and three other clusters. Relative frequency was shown as a bar graph on the right. **c** Boxplots demonstrating the frequencies of the CD4^+^ and CD8^+^ T cell clusters among DCB and NDB patients before and after immunotherapy. **d** Paired PBMC samples analysis before and after immunotherapy demonstrating the changes in frequencies of the CD8^+^ T cell clusters among DCB and (**e**) NDB patients. *p < 0.05. NSCLC non-small cell lung cancer, t-SNE t-distributed Stochastic Neighbor Embedding, PBMCs peripheral blood mononuclear cells, DCB durable clinical benefit, NDB no durable clinical benefit, DC dendritic cells, pDC plasmacytoid dendritic cells, DNT double-negative T cells, DPT double-positive T cells, NK cells natural killer cells.

Using dimensionality reduction t-SNE analysis, we further characterized the phenotypes of these 12 major immune cell clusters, and revealed 37 immune cell clusters (Fig. 2a). A heatmap of the normalized mean expression of 42 membranous or intracellular markers used to identify the 12 major immune cell clusters is shown in Fig. 2b. Generally, we identified one cluster in DC, pDC, basophils, DNT, DPT, and γδT cells; three in B cells; four in NK cells; seven in monocytes; seven in CD4^+^ T cells; seven in CD8^+^ T cells; and three other clusters (Fig. 2b). The frequencies of immune cell clusters subsets between the two groups before and after immunotherapy was compared (Fig. 2c; Supplementary Fig. 3a). Before immunotherapy, the frequency of cluster 36 (CD8^+^ T cells) in the DCB group was significantly higher than that in the NDB group (*p* = 0.034; Fig. 2c), whereas the frequency of cluster 32 (NK cells) was significantly lower (*p* = 0.016; Supplementary Fig. 3a). After treatment, the frequencies of clusters 2 (B cells; *p* = 0.043; Supplementary Fig. 3a) and 35 (CD8^+^ T cells; *p* = 0.022; Fig. 2c) cells were higher in the DCB group than in the NDB group. Next, we performed a paired analysis of samples from 14 patients with paired samples (DCB, *n* = 6; NDB, *n* = 8) before and after immunotherapy. In the DCB group, the frequencies of clusters 35 and 36 increased for all patients after treatment compared to baseline (paired *t*-test, *p* = 0.071 and *p* = 0.08, respectively), whereas they decreased for some patients in the NDB group (paired *t*-test, *p* = 0.53, *p* = 0.59, respectively; Fig. 2d, e); regrettably, differences were not significant. To further reveal the heterogeneity of T cell clusters, we examined the expression of functional markers to identify T cell subpopulations (Fig. 2b). Cluster 36 showed a higher expression of Granzyme B, T-bet, and CD57 than other T-cell clusters. Therefore, we hypothesized that cluster 36 at baseline might be a biomarker of the response to immunotherapy in NSCLC.

### CD57^+^CD8^+^ T cells were predictive for response to anti-PD-1 treatment in patients with NSCLC

To further validate our findings, we conducted a prospective study using two separate cohorts that were independent of the CyTOF cohort. Cohort 1 comprised 27 NSCLC patients (16 in the DCB group and 11 in the NDB group) treated with PD-1 inhibitor monotherapy between May 2021 and April 2022. Cohort 2 consisted of 48 NSCLC patients (27 in the DCB group and 21 in the NDB group) treated with a combination of immunotherapy and platinum-based chemotherapy between May 2021 and December 2022. All PBMCs were collected before immunotherapy. Detailed clinical information was presented in Table 2.

**Table 2:** The clinical characteristics of advanced NSCLC in flow cytometry cohorts (*n* (%)).

Characteristics	Cohort 1 (*n* = 27)	Cohort 2 (*n* = 48)	*p* value
Gender			0.574
Male (M)	23 (85.2)	43 (89.6)	
Female (F)	4 (14.8)	5 (10.4)	
Age (years)			0.123
<65	8 (29.6)	23 (47.9)	
≥65	19 (70.4)	25 (52.1)	
Smoking			0.520
YES	22 (81.5)	36 (75.0)	
NO	5 (18.5)	12 (25.0)	
Line of therapy			0.083
First line	15 (55.6)	36 (75.0)	
Further line	12 (44.4)	12 (25.0)	
Histological type			0.079
LUAD	13 (48.1)	24 (50.0)	
LUSC	14 (51.9)	17 (35.4)	
Others	0 (0.0)	7 (14.6)	
Tumor stage			0.404
Stage IIIB/C	5 (18.5)	13 (27.1)	
Stage IV	22 (81.5)	35 (82.9)	
Clinical response			0.800
DCB	16 (59.3)	27 (56.25)	
NDB	11 (40.7)	21 (43.75)	

*NSCLC* non-small cell lung cancer, *LUAD* lung adenocarcinoma, *LUSC* lung squamous cell carcinoma, *DCB* durable clinical benefit, *NDB* no durable clinical benefit.

**Fig. 3:**
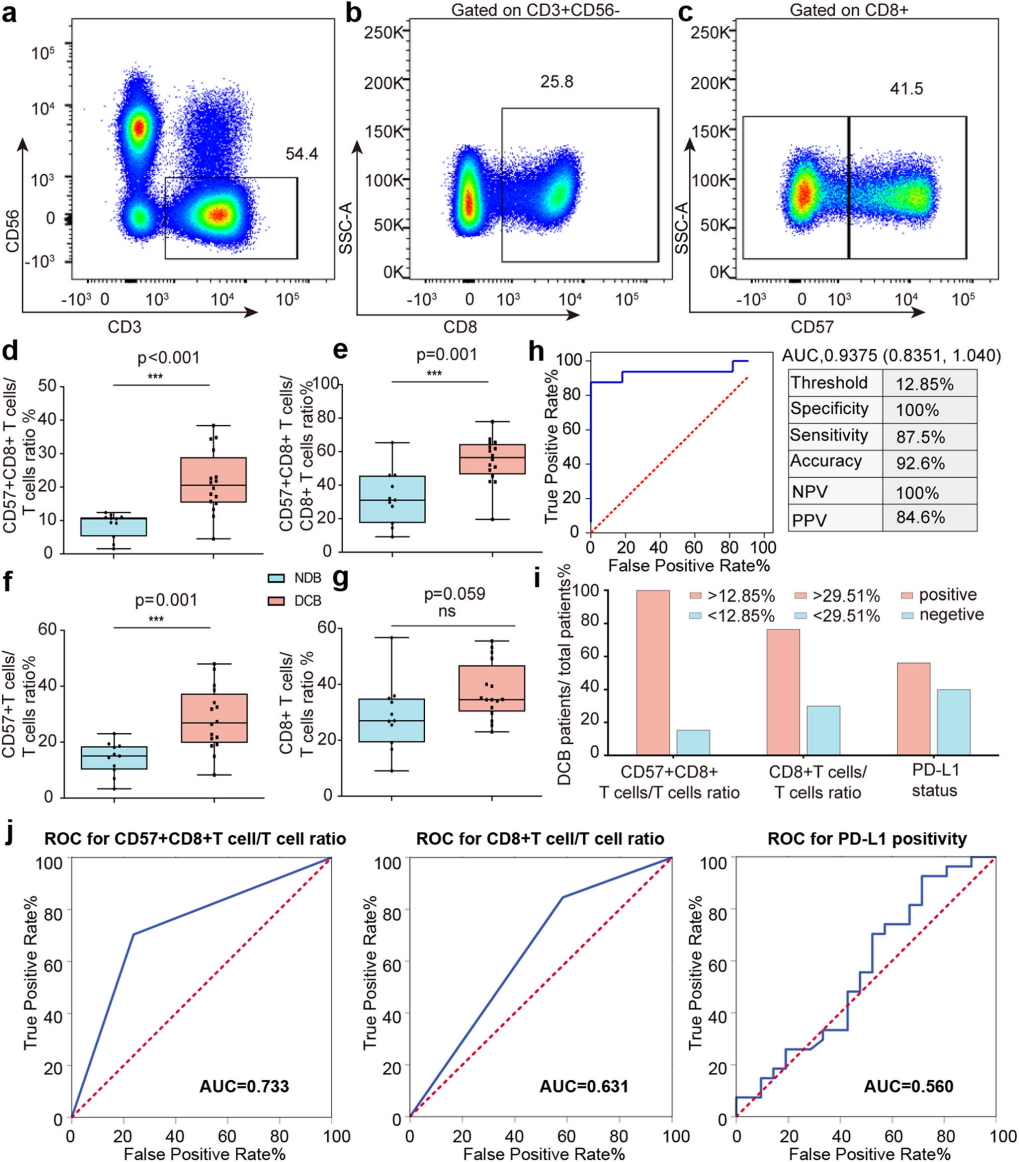
Flow cytometry-based quantification of CD57^+^CD8^+^T cells predicts immunotherapeutic response in two prospective NSCLC cohorts. **a**–**c** Flow cytometry-based quantification of T cells, CD8^+^ T cells, CD57^+^CD8^+^ T cells and CD57^-^CD8^+^ T cells in the prospective NSCLC cohorts. **d** CD57^+^CD8^+^ T cells/T cells ratio (*p* < 0.001), **e** CD57^+^CD8^+^ T cells/ CD8^+^ T cells ratio (*p* = 0.001) and **f** total CD57^+^ T cells/T cells (*p* = 0.001) as determined by flow cytometry predicted clinical response to immunotherapy (*n* = 27). **g** Total CD8^+^ T cells/T cells ratio was not of predictive value (*p* = 0.059, *n* = 27). **h** ROC curve analyzed the ability of CD57^+^CD8^+^ T cells/T cells ratio to identify responders (AUC = 0.9375, *n* = 27). Sensitivity (87.5%) refers to the proportion of true positive subjects with the disease among all subjects with disease. Specificity (100.0%) refers to the proportion of true negative subjects without the disease among subjects without disease. PPV (84.6%) refers to the proportion of patients with positive results among subjects with positive results. NPV (100.0%) refers to the proportion of subjects without disease with a negative result among subjects with negative results. Accuracy (92.6%) refers to the proportion of subjects correctly classified among all subjects. **i** DCB proportion comparing the ability of CD57^+^CD8^+^ T cells/T cells ratio as determined by flow cytometry, total CD8^+^ T cells/T cells ratio by flow cytometry, and PD-L1 status by conventional IHC to predict treatment response. **j** ROC curves for predicting treatment response using the CD57^+^CD8^+^ T cells/T cells ratio (AUC = 0.733), CD8^+^ T cells/T cells ratio (AUC = 0.631), and PD-L1 positivity (AUC = 0.560) (*n* = 48). ***p ≤ 0.001. NSCLC non-small cell lung cancer, ROC receiver operating characteristic, AUC area under the curve, PPV positive predictive value, NPV negative predictive value, DCB durable clinical benefit, PD-L1 programmed death-ligand 1, IHC immunohistochemistry.

To test our hypothesis, we conducted flow cytometry analysis on PBMC samples obtained from both cohort 1 and 2 (Fig. 3a–c, Supplementary Fig. 4a). In cohort 1, patients in DCB group had a significantly higher ratio of CD57^+^CD8^+^ T cells to T cells compared to patients in the NDB group (21.39% ± 9.29% vs. 8.67% ± 3.73%, *p* < 0.001; Fig. 3d). Similarly, the DCB group showed significantly higher ratios of CD57^+^CD8^+^ T cells to CD8^+^ T cells (54.70% ± 13.61% vs. 32.53% ± 16.18%, *p* = 0.001; Fig. 3e) and CD57^+^ T cells to total T cells (28.24% ± 11.26% vs. 14.19% ± 5.81%, *p* = 0.001; Fig. 3f) compared to the NDB group. These data suggested that upregulated CD57 expression in both CD8^+^ T and T cells can predict the response to anti-PD-1 immunotherapy in patients with NSCLC.

While previous studies have shown that CD8^+^ T cells, along with other biomarkers such as PD-L1 expression, tumor mutation burden (TMB), and human leukocyte antigen (HLA) class I expression, can predict response to ICIs in patients with NSCLC^25–27^, our study demonstrated that the ratio of CD8^+^ T cells to total T cells alone was not able to predict the response (37.65% ± 9.88% vs. 28.63% ± 12.38%, *p* = 0.059; Fig. 3g). Receiver operating characteristic (ROC) curve analysis revealed a strong correlation between the CD57^+^CD8^+^ T cells to total T cells ratio and the response status (area under the curve (AUC): 0.9375; Fig. 3h). Using a cutoff threshold of 12.85% CD57^+^CD8^+^ T cells of the total T cells, we achieved 92.6% accuracy, 100% specificity, and 87.5% sensitivity in predicting the response to anti-PD-1 immunotherapy (Fig. 3h). In our cohort, the CD57^+^CD8^+^ T cells to total T cells ratio was the only parameter that showed significant predictive value, while other clinicopathological parameters, including CD8^+^ T cells to total T cells ratio and PD-L1 positivity, did not reach statistical significance (Table 3). Furthermore, using the aforementioned cutoff value (12.85%), patients were stratified into high and low CD57^+^CD8^+^ T cells/T cells ratio groups, and none of the clinicopathological parameters were correlated with the CD57^+^CD8^+^ T cells/T cells ratio (Supplementary Table 3). To further support these results, we observed that all patients with a CD57^+^CD8^+^ T cells to total T cells ratio above 12.85% achieved a durable clinical benefit, while only 15.4% of patients with a ratio below 12.85% exhibited a durable clinical benefit (Fig. 3i). However, the CD8^+^ T cells/T cells ratio and PD-L1 status did not demonstrate the same level of accuracy in predicting the response in our cohort (Fig. 3i).

**Table 3:** Results of binomial logistic regression analysis to stratify DCB and NDB patients.

Characteristics	OR (95%CI)	*P* value
CD57^+^CD8^+^ T cells/T cells ratio (%)	0.666 (0.448, 0.001)	0.045*
CD8^+^ T cells/T cells ratio (%)	1.021 (0.838, 1.245)	0.834
PD-L1 status		
Negative	Reference	
Positive	0.950 (0.050, 18.042)	0.974

*p *<* 0.05*, DCB* durable clinical benefit, *NDB* no durable clinical benefit, *PD-L1* Programmed death-ligand 1.

The bold value of 0.045 is highlighted to emphasize the significant difference in the CD57+CD8+ T cells/Tcells ratio when distinguishing between patients with DCB and NDB.

To assess the predictive efficacy of the cutoff threshold of 12.85% CD57^+^CD8^+^ T cells among total T cells in a broader context, we conducted validation using cohort 2. Consistent with our initial cohort findings in cohort 1, the cutoff threshold of 12.85% CD57^+^CD8^+^ T cells/T cells ratio demonstrated its effectiveness in distinguishing patients who achieved DCB from those who did not (NDB) in the validation dataset. The AUC value for predicting clinical response was 0.733 (Fig. 3j).

Importantly, when compared to the conventional biomarker PD-L1 (AUC = 0.631) and the CD8^+^ T cells to total T cells ratio (AUC = 0.560), the CD57^+^CD8^+^ T cells to total T cells ratio exhibited even stronger clinical relevance (Fig. 3j). These findings highlighted the potential of CD57^+^CD8^+^ T cells/T cells ratio in accurately predicting the response to immunotherapy, surpassing the performance of the traditional PD-L1 biomarker. These results further supported the notion that CD57^+^CD8^+^ T cells have significant potential for evaluating the clinical efficacy of immunotherapy in a broader patient population.

### CD57^+^CD8^+^T cells, as determined using mIHC/IF, predicted response to anti-PD-1 treatment in patients with NSCLC

To further investigate the hypothesis in tissue samples, we collected 90 pre-treatment Formalin-Fixed Paraffin-Embedded (FFPE) tissues. These included all archived pre-treatment FFPE tissues from the patients who had their blood collected, as well as tissues from other patients. Overall, 90 FFPE samples were obtained, and the response statuses of these patients were categorized into DCB (*n* = 44) and NDB (*n* = 46). Detailed clinical information was presented in Supplementary Table 4.

**Fig. 4:**
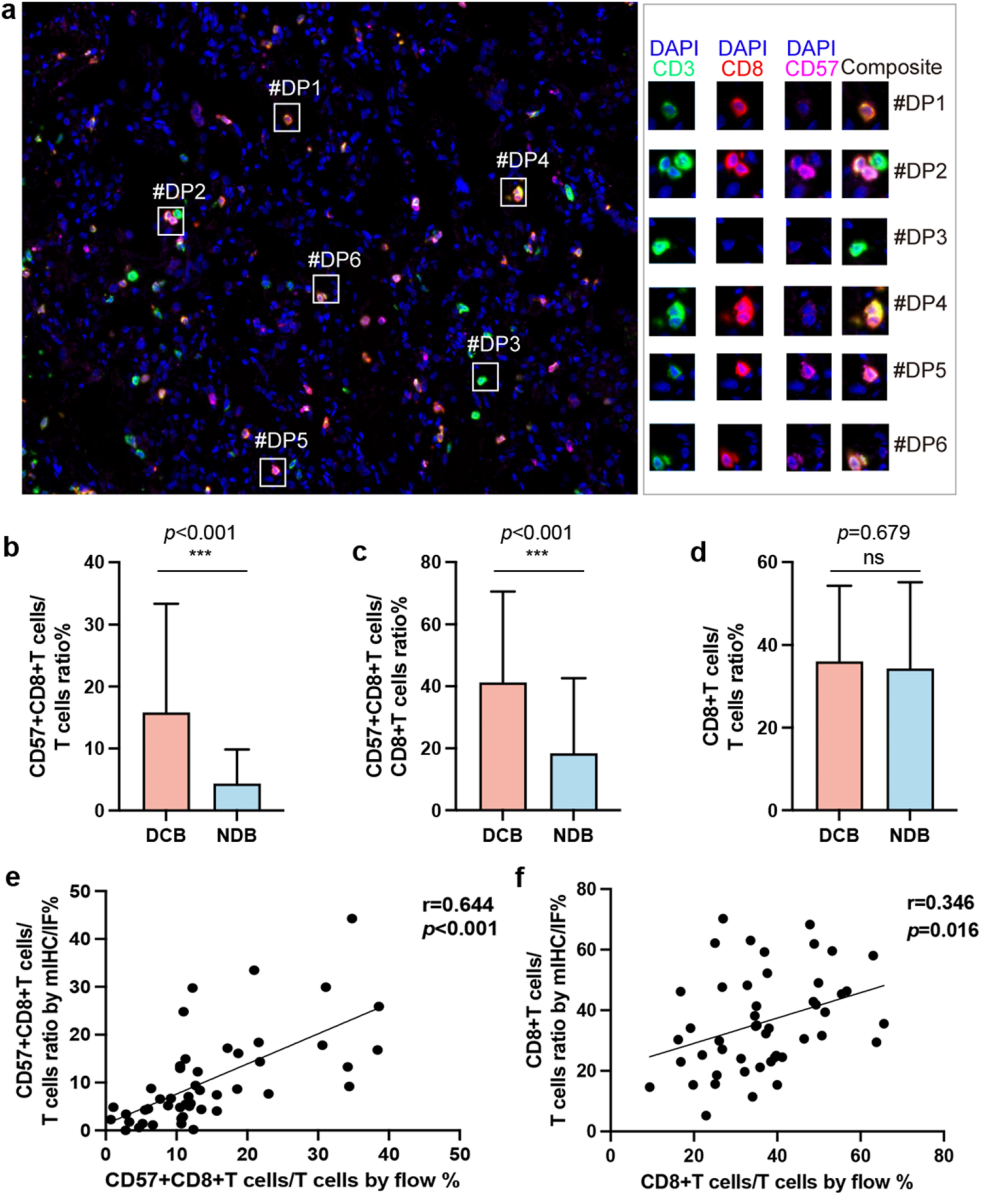
mIHC/IF-based quantification of CD57^+^CD8^+^ T cells predicts immunotherapeutic response in a retrospective NSCLC cohort (*n* = 90). **a** Representative images of NSCLC tissue stained using mIHC/IF [CD3 (green), CD8 (red), CD57 (pink), DAPI (blue)]. **b** mIHC/IF-based CD57^+^CD8^+^ T cells/T cells ratio (*p* < 0.001) and (**c**) CD57^+^CD8^+^ T cells/CD8^+^ T cells ratio (*p* < 0.001) predicted immunotherapeutic response in a retrospective NSCLC cohort (*n* = 90). **d** Total CD8^+^ T cells/T cells ratio by mIHC/IF was not of predictive value to immunotherapy (*p* = 0.679, *n* = 90). **e** Spearman’s rank correlation analysis was performed to compare the CD57^+^CD8^+^ T cells/T cells ratio (*r* = 0.644, *p* < 0.001), and **f** CD8^+^ T cells/T cells ratio (*r* = 0.346, *p* = 0.016) in blood and tumor samples (*n* = 48). ***p < 0.001. mIHC/IF multiplex immunohistochemistry/immunofluorescence, NSCLC non-small cell lung cancer.

We developed a 3-marker mIHC/IF panel for patients with NSCLC and analyzed FFPE specimens (Fig. 4a). We compared the ratios of CD57^+^CD8^+^ T cells to other T cell subsets between the DCB and NDB patient groups. The results demonstrated that patients in the DCB group had significantly higher CD57^+^CD8^+^ T cells to total T cells ratio (15.83% ± 17.48% vs. 4.34% ± 5.52%, *p* < 0.001) and CD57^+^CD8^+^ T cells to CD8^+^ T cells ratio (41.24% ± 29.29% vs. 18.35% ± 24.29%, *p* < 0.001) compared to the NDB group (Fig. 4b, c). However, the CD8^+^ T cells to total T cells ratio was not able to predict the treatment response (36.02% ± 18.28% vs. 34.30% ± 20.81%, *p* = 0.679; Fig. 4d).

Furthermore, we observed a significant correlation between the frequency of CD57^+^CD8^+^ T cells/T cells ratio in tumor tissues and that in peripheral blood (*r* = 0.644, *p* < 0.001; Fig. 4e). This finding suggested that the trend of CD57^+^CD8^+^ T cells/ T cells ratio in tumor tissues was consistent with that in peripheral blood, indicating that the measurement of CD57^+^CD8^+^ T cells/T cells ratio in blood may be used as a predictive marker for ICI responders.

To further investigate the homogeneity of CD57^+^CD8^+^ T cells between blood and tumor tissue, we utilized publicly available datasets from the Gene Expression Omnibus (GEO) database. We selected data from four patients included single-cell transcriptomic and T-cell receptor (TCR) sequencing data from both blood and tissue compartments for our analysis (Supplementary Fig. 5). By integrating the transcriptomic and TCR sequencing data, we conducted an analysis to assess the TCR homogeneity between CD57^+^CD8^+^ T cells in blood and tissue. In the four patients analyzed, except for patient 2, there was a consistent presence of identical TCR clones in both the blood and tissue samples of CD57^+^CD8^+^ T cells (Supplementary Fig. 5d). This finding suggested the presence of shared clonal populations of CD57^+^CD8^+^ T cells between these two compartments, indicating the potential migration of CD57^+^CD8^+^ T cell populations from blood to tissue.

### Transcriptomic analysis revealed differences between CD57^+^CD8^+^ T and CD57^-^CD8^+^ T cells

**Fig. 5:**
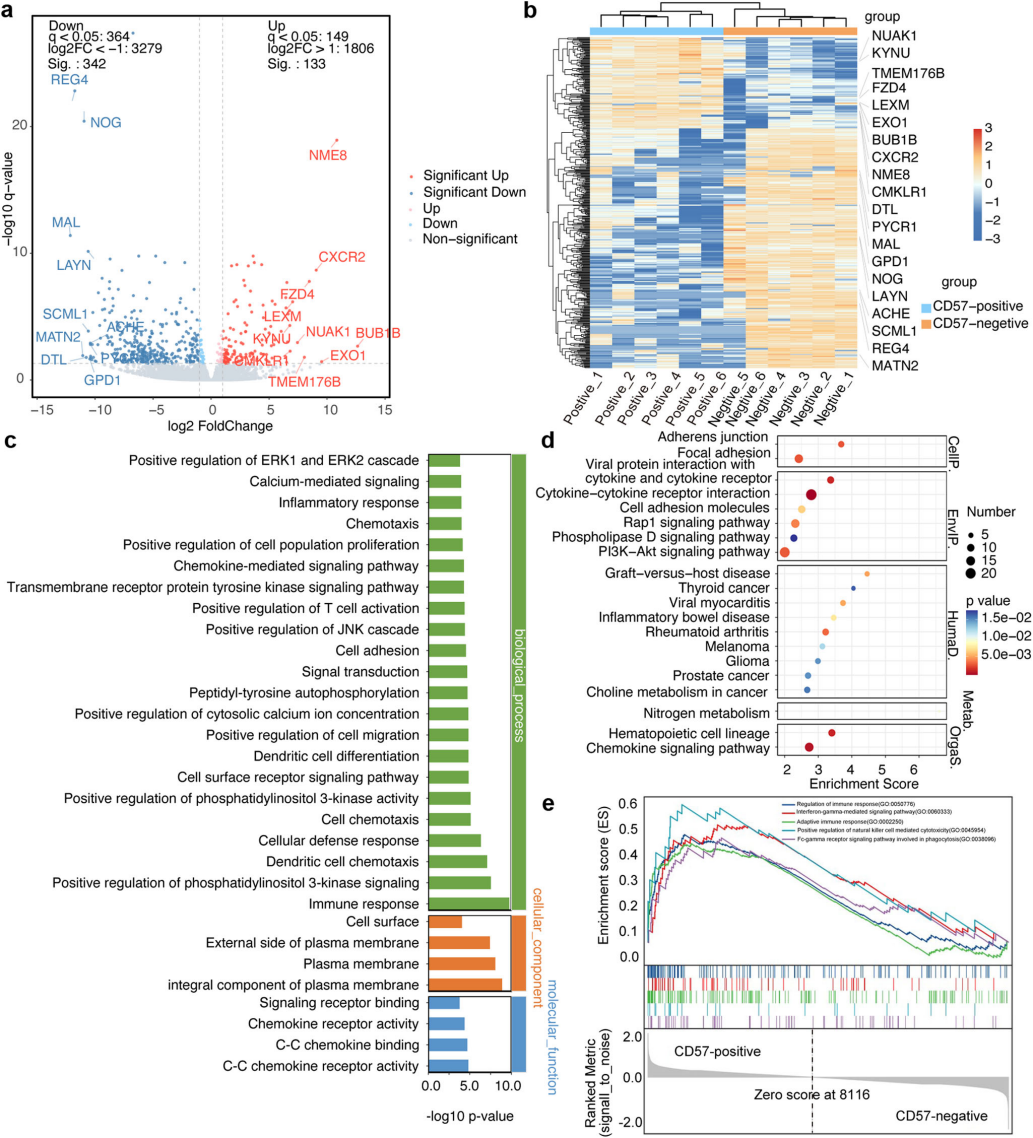
Identification of DEGs and screening of genes-based GO, KEGG and GSEA analysis between CD57^+^CD8^+^ T cells and CD57^-^CD8^+^ T cells in patients with NSCLC. **a** Volcano plot showing the 475 DEGs between CD57^+^CD8^+^ T cells and CD57^−^CD8^+^ T cells, including 133 upregulated genes and 342 downregulated genes. Red and blue colors represent upregulated and downregulated genes, respectively. **b** Clustering analysis of DEGs and samples. The color scale bar for heat intensity indicates Log2(Fold Change). Columns, samples; rows, DEGs. The samples were grouped into two distinct clusters: CD57^+^CD8^+^ T cell cluster and CD57^-^CD8^+^ T cell cluster. **c** GO analysis of DEGs. The most enriched 30 GO terms in biological process, cellular component, and molecular function. The *y* axis represents GO terms and the *x* axis represents the value of -log10 (*p*-value). **d** KEGG enrichment analysis of DEGs. The x axis represents enrichment score and the y axis represents pathway. Size and color of the bubble represent the amount of DEGs enriched in pathway and enrichment significance, respectively. **e** Representative GSEA results showing enrichment of the immune-associated pathways in CD57^+^CD8^+^ T cells. DEGs differentially expressed genes, GO Gene Ontology, KEGG Kyoto Encyclopedia of Genes and Genomes, GSEA Gene Set Enrichment Analysis, NSCLC non-small cell lung cancer.

To gain further insights into the molecular mechanisms of CD57^+^CD8^+^ T cells, we performed RNA-seq analysis. We enrolled six patients with NSCLC who received single-agent PD-1 inhibitors from the flow cytometry cohort. Among these patients, three belonged to the DCB group, and the remaining three belonged to the NDB group. For RNA-seq analysis, we sorted both CD57^+^CD8^+^ T and CD57^−^CD8^+^ T cells from PBMCs before immunotherapy (Supplementary Fig. 4b). We aimed to identify DEGs between the two cell populations. Our analysis revealed a total of 475 DEGs, with 133 genes upregulated and 342 genes downregulated in CD57^+^CD8^+^ T cells compared to CD57^−^CD8^+^ T cells (Fig. 5a). Clustering analysis of gene expression clearly separated the data into two clusters (CD57^+^CD8^+^ T and CD57^-^CD8^+^ T cell clusters), showing the distinct transcriptomic profiles between CD57^+^CD8^+^ T and CD57^−^CD8^+^ T cells (Fig. 5b). These findings indicated that CD57^+^CD8^+^ T cells have distinct gene expression profiles compared to CD57^−^CD8^+^ T cells. The identified DEGs provided valuable information for understanding the molecular characteristics and potential functional roles of CD57^+^CD8^+^ T cells in the context of PD-1 inhibitor treatment in NSCLC patients.

Gene Ontology (GO), Kyoto Encyclopedia of Genes and Genomes (KEGG), and Gene Set Enrichment Analysis (GSEA) analyses were performed to analyze the function of DEGs. In our study, 460 DEGs were mapped to the GO database, and the top 30 significantly enriched GO terms were shown in Fig. 5c. The analysis revealed that the majority of the DEGs were associated with immune-related biological process, including immune response, chemokine receptor activity, and positive regulation of T cell activation. Subsequently, 209 DEGs could be annotated by the KEGG database. Based on the top 20 pathway enrichment analyses, DEGs were significantly enriched in immune-related signaling pathways, including the cytokine-cytokine receptor interaction, chemokine signaling pathway, and PI3K-Akt signaling pathway (Fig. 5d). GSEA further revealed that the CD57^+^CD8^+^ T cell cluster was significantly involved in immune-associated pathways, such as regulating the immune response, interferon-gamma-mediated signaling pathway, adaptive immune response, positive regulation of natural killer cell-mediated cytotoxicity, and the Fc-gamma receptor signaling pathway involved in phagocytosis (Fig. 5e).

These findings collectively suggested that the DEGs identified in CD57^+^CD8^+^ T cells were predominantly involved in immune-related processes and signaling pathways. This supported the notion that CD57^+^CD8^+^ T cells played a crucial role in immune responses and potentially contributed to the therapeutic response to PD-1 inhibitors in NSCLC.

Next, we conducted further analysis to explore potential qualitative differences in CD57^+^CD8^+^ T cells between DCB and NDB patients. We identified a total of 47 DEGs, with 40 genes upregulated and 7 genes downregulated in CD57^+^CD8^+^ T cells in the DCB group compared to the NDB group (Supplementary Fig. 6a). Furthermore, the clustering analysis of gene expression effectively segregated the data into two distinct clusters, namely the DCB cluster and the NDB cluster, thereby indicating discernible transcriptomic profiles between the DCB and NDB groups (Supplementary Fig. 6b). To gain insights into the functional implications of the DEGs, we performed GO and KEGG analyses. However, the outcomes of our analyses (Supplementary Fig. 6c, d) revealed that the majority of the DEGs were not primarily associated with immune-related processes. Consequently, these findings suggested that the observed differences in CD57^+^CD8^+^ T cells between DCB and NDB patients were primarily quantitative in nature, reflecting variations in abundance rather than qualitative distinctions.

In summary, our analysis has elucidated that the differences observed in CD57^+^CD8^+^ T cells between DCB and NDB patients are primarily quantitative. The transcriptomic profiles and functional analysis have successfully demonstrated distinct patterns in gene expression, thereby highlighting the significance of quantitative differences in CD57^+^CD8^+^ T cells within the context of DCB and NDB treatments.

## Discussion

Currently, ICIs are widely used and have made significant advances in treating patients with NSCLC. However, ICIs are ineffective in most patients. Despite the availability of biomarker stratification, clinical responses differ. In this context, there is significant interest in detecting potential biomarkers to precisely identify patients with DCB and NDB before immunotherapy initiation.

In our study, using a high-dimensional single-cell CyTOF method combined with clustering analyses, we investigated differential immune signatures of PBMCs in patients with DCB and NDB before and 12 weeks after anti-PD-1 immunotherapy. We found that CD57^+^CD8^+^ T cells were the strongest predictors of responsiveness to anti-PD-1 immunotherapy. However, CD8^+^ T cells alone could not predict this response. Several studies have reported that CD8^+^ T cells, combined with other signatures, including PD-L1 expression, TMB, and HLA class I expression, can predict response to ICIs in patients with NSCLC^25–27^. These observations demonstrated that the predictive value of CD8 expression alone was limited; however, a further refined subpopulation of CD8^+^ T cells (i.e., CD57^+^CD8^+^ T cells) might predict the response to immunotherapy.

Next, using a different method (flow cytometry) in an independent validation cohort, we confirmed that CD57^+^CD8^+^ T cells were associated with a good response, whereas PD-L1 was not a valuable prognostic biomarker, even if a trend was observed. Subsequently, we further quantified CD57^+^CD8^+^ T cells in tumor tissues using the mIHC/IF method, suggesting that the frequency trend of CD57^+^CD8^+^ T cells in tumor tissues was consistent with that in peripheral blood, which could be used to predict the anti-PD-1 response. Previously, based on a meta-analysis of 26 published studies with 7656 patients, Hu et al. investigated the prognostic role of tumor-infiltrating CD57^+^ lymphocytes in solid tumors and found that an increase in CD57^+^ lymphocyte infiltration significantly improved overall survival and disease-free survival^28^. Solid tumors with a high density of intratumoral CD57^+^ lymphocytes showed an inverse correlation with lymph node metastasis and Tumor, Node, and Metastasis stage. This finding was consistent with the previous results. CyTOF results showed that CD57^+^CD8^+^ T cells in the peripheral blood expressed CD45RA, T-bet, and Granzyme B; however, CD27, CD28, and CCR7 were absent. These results were consistent with the study conducted by Brenchley et al., who also reported the absence of CD27, CD28, and CCR7 expression in CD57^+^CD8^+^ T cells^29^. We speculated that a possible explanation for this observation was the high expression of cytokines and cytotoxic molecules, including Granzyme B and T-bet, in CD57^+^ CD8^+^ T cells. This hypothesis was supported by previous studies, which demonstrated that CD57^+^CD8^+^ T cells displayed a late-differentiated T-cell phenotype with enhanced cytotoxicity and effector functions^30^. Previous studies have linked ICI response to an increase in T cells with late differentiation status^21,31^. NSCLC treatment response was associated with CD45RA-expressing T effector memory cells^32^. These results were consistent with the conclusions of this study.

RNA-seq was used in our study, and 475 DEGs from CD57^+^CD8^+^ T cells to CD57^-^CD8^+^ T cells were identified. Functional analysis of the DEGs using the GO and KEGG databases revealed that DEGs were significantly enriched in immune-related signaling pathways. This might explain, at least in part, our results regarding the positive correlation between CD57^+^CD8^+^ T cells and the clinical response to anti-PD-1 immunotherapy. This was consistent with a previous study showing that CD57^+^CD8^+^ T cells are associated with neoantigen-specific CD8^+^ T cells^33^. Thus, CD57^+^CD8^+^ T cells were a useful biomarker for the response to anti-PD-1 immunotherapy and can be a valuable complementary approach to further compensate for the insufficiency of only testing for PD-L1 expression and improve the efficacy of immunotherapy.

While our study focused on the CD3^+^CD8^+^CD57^+^ and CD3^+^CD8^+^CD57^-^ subsets, it is important to acknowledge the potential influence and confounding effects of the CD56^+^ subset in our experimental system. CD56^+^ cells, including NKT-like cells, have been implicated in immune regulation and gene expression modulation^34,35^. They possess unique functional properties and can interact with other immune cell subsets, such as CD8^+^ T cells. The presence of CD56^+^ cells in our sorted subsets could potentially influence the transcriptomic profiles and functional outcomes observed. However, it is important to note that in this study, we did not specifically investigate the role of CD56^+^ cells or perform functional characterization of these subsets. Consequently, the specific contribution of CD56^+^ cells to the observed transcriptomic changes remains speculative and warrants further investigation. Future studies that specifically address the functional role of CD56^+^ cells, such as depletion or enrichment experiments, would provide valuable insights into their potential influence on the observed gene expression profiles in the CD3^+^CD8^+^CD57^+^ and CD3^+^CD8^+^CD57^-^ subsets. Additionally, single-cell RNA-seq approaches may allow for a more comprehensive characterization of the cellular heterogeneity within these subsets and help elucidate the potential interactions and functional implications of CD56^+^ cells.

Our study had some limitations. First, this cohort’s heterogeneous nature comprised patients treated with different immunotherapeutic agents. Second, this study conducted only the phenotypic and transcriptional analyses and did not evaluate the functional aspects. Thus, multicenter confirmatory studies with larger and independent cohorts are required to validate our observations. Furthermore, future studies should incorporate functional assays to investigate the cytokine production and cytotoxicity of the CD57^+^CD8^+^ population in response to therapy, along with exploring potential pathways involved. These functional assays will provide a more comprehensive understanding of the functional capabilities of CD57^+^CD8^+^ T cells and their contribution to therapy response.

In conclusion, our study demonstrates that both blood- and tissue-based measurements of CD57^+^CD8^+^ T cells may serve as promising biomarkers for predicting the response to anti-PD-1 treatment in NSCLC. However, further studies are required to validate our observations, and additional experiments are required to explore potential mechanisms.

## Methods

### Study design and patient samples

The study included patients with advanced NSCLC who were treated with PD-1 inhibitors at The First Affiliated Hospital, College of Medicine, Zhejiang University (China). Patients diagnosed with clinical stage IIIB/IIIC enrolled in this study were deemed inoperable by the lung multidisciplinary team and suitable for immunotherapy. Pathological or clinical staging was performed according to the eighth edition of the American Joint Committee on Cancer guidelines. Treatment response was investigator-assessed based on the Response Evaluation Criteria in Solid Tumors version 1.1. Response to immunotherapy was classified into a DCB (complete response, partial response, or stable disease (SD) lasting >6 months) and NDB (progressive disease or SD lasting <6 months). The CyTOF cohort included 20 patients who received single-agent PD-1 inhibitors between August 2019 and July 2020. Blood samples were collected before treatment initiation and approximately 12 weeks after the initiation of anti-PD-1 immunotherapy. Flow cytometry analysis was performed in two separate cohorts. Cohort 1 consisted of 27 NSCLC patients treated with single-agent PD-1 inhibitors between May 2021 and April 2022. Cohort 2 included 48 NSCLC patients treated with a combination of immunotherapy and platinum-based chemotherapy between May 2021 and December 2022. Blood samples were collected from both cohorts before the initiation of anti-PD-1 immunotherapy. For the study, we retrospectively obtained FFPE tissue sections. These samples were archived in the pathology department at the First Affiliated Hospital, College of Medicine, Zhejiang University. The FFPE samples were originally collected from patients who underwent surgical resection or biopsy procedures at the hospital before receiving immunotherapy. In total, 90 patients were included in this analysis, and all FFPE samples underwent pathological examination.

### Isolation of human PBMCs

For PBMC isolation, we collected 10 ml fresh whole blood from patients with NSCLC in K2EDTA-coated vacutainer tubes (BD Biosciences). Ficoll-Paque PLUS (GE Healthcare) was used to separate PBMCs by density gradient centrifugation. Subsequently, washes were performed with the FACS buffer (PBS + 0.5% bovine serum albumin) twice at 400 g for 10 min at room temperature. PBMCs were resuspended in the FACS buffer and counted.

### CyTOF staining, data acquisition, and analysis

CyTOF data were collected and analyzed by PLTTech Inc. (Hangzhou, China). We selected 42 markers of interest based on previous studies on NSCLC. The MaxPAR antibody Labeling kit (Fluidigm) was used to label antibodies with mass tags; detailed information is presented in Supplementary Table 1. Each metal-conjugated antibody was titrated to obtain the optimal concentration. To distinguish live from dead cells, obtained PBMCs were first stained for 5 min with 100 μL of cisplatin (250 nM, Fluidigm). After incubating in Fc receptor-blocking solution for 20 min, the PBMCs were stained with a surface antibody cocktail for 30 min on ice. Next, a 200 μL intercalation solution (maxpar Fix and Perm Buffer containing 250 nM 191/193Ir, Fluidigm) was used to fix the PBMCs overnight after which they were stained with an intracellular antibody cocktail for 30 min on ice. After washing, PBMCs were stained with a unique barcode isotope combination for 30 min to label individual cell samples. Finally, the PBMCs were washed and resuspended in deionized water, added to 20% EQ beads (Fluidigm), and data were obtained using a mass cytometer (Helios, Fluidigm).

For each sample, raw data was debarcoded using a doublet-filtering scheme with mass-tagged barcodes. The bead normalization method was used to normalize data from different batches^36^. Next, live and single immune cells were acquired by manual gating using FlowJo software. The X-shift clustering algorithm^37^ was used to determine cell phenotypes based on the level of marker expression. On a heat map of clusters versus markers, cell types were annotated according to their marker expression. Furthermore, a dimensionality reduction algorithm, t-SNE^38^, was used to visualize the high-dimensional data in two dimensions and show each cluster’s distribution, marker expression, and differences between the groups or samples. The frequency of annotated cell populations was evaluated using a t-test.

### Flow cytometry

All antibodies used for flow cytometry were obtained from BioLegends. PBMCs were incubated with the following Ab-conjugates on ice in the dark: anti-CD66b-PE-Cy7 (G10F5, 305115, 1:100), anti-CD3-BV510 (UCHT1, 300447, 1:100), anti-CD56-BV711 (HCD56, 318335, 1:100), anti-CD8a-APC-Cy7 (RPA-T8, 301015, 1:100), anti-CD57-PB (HCD57, 322315, 1:100). Incubation was completed for 30 min, then samples were washed twice with FACS buffer and resuspended in 300 μL of the same buffer. Furthermore, 7-AAD (BioLegend, 420404, 1:100) was added to PBMCs immediately before analysis. The flow cytometry was carried out using a BD FACSFortessa Multicolor Flow Cytometer (BD Biosciences). The CD57^+^CD8^+^T cells/T cells ratio was calculated using the following formula: $\frac{{\rm{CD}}57+{\rm{CD}}8+{\rm{CD}}3+{\rm{cells}}}{{\rm{CD}}3+{\rm{cells}}}100 \%$. The CD57^+^CD8^+^ T cells/CD8^+^ T cells ratio was calculated using the formula below: $\frac{{\rm{CD}}57+{\rm{CD}}8+{\rm{CD}}3+{\rm{cells}}}{{\rm{CD}}8+{\rm{CD}}3+{\rm{cells}}}100 \%$. As well, the total CD8^+^ T cells/T cells ratio and the total CD57^+^ T cells/T cells ratio were calculated as the proportion of CD8^+^CD3^+^ cells among total CD3^+^ cells and the proportion of CD57^+^CD3^+^ cells among total CD3^+^ cells, respectively.

### mIHC/IF staining

The mIHC/IF staining was performed with an Opal Polaris 7-Color Multiplex IHC kit from Akoya Biosciences. Briefly, each FFPE tissue section was baked at 65 °C for 1 h. After deparaffinization, rehydration, and microwave antigen repair, the slides were blocked (Akoya Biosciences, USA), and incubated with primary antibodies against CD3 (D7A6E, CST, 85061 T, 1:200), CD57 (HNK-1, CST, 72031 S, 1:200), and CD8 (EPR22483-288, Abcam, ab245118, 1:500), respectively, followed by incubation with Opal Polymer HRP Ms+Rb (Akoya Biosciences, USA). The slides were then incubated with Opal Fluorophore-conjugated tyramide signal amplification reagent (Akoya Biosciences, USA). After signal amplification, microwave antigen repair was performed to remove the detected antibodies. This process was repeated using another Opal Fluorophore. The above steps were repeated until the slides were labeled with all antibodies and DAPI. Finally, the slides were sealed with an anti-fluorescence quencher. Visualization of slides was done using Vectra Polaris Quantitative Pathology Imaging Systems (Akoya Biosciences, USA); analysis and scoring were performed using inForm software (Akoya Biosciences, USA). The CD57^+^CD8^+^ T cells/T cell ratio, CD57^+^CD8^+^ T cells/ CD8^+^ T cells ratio, and total CD8^+^ T cells/T cells ratio were calculated the same as flow cytometry.

### IHC staining

Tumor PD-L1 expression was assessed through IHC on FFPE sections, employing an anti-PD-L1 monoclonal antibody (clone 22C3; Dako, USA). PD-L1 expression scores were reported as the percentage of tumor cells with membranous staining as determined by pathologists. PD-L1 subgroups were defined as negative (PD-L1 < 1%), and positive (PD-L1 ≥ 1%).

### Fluorescence-activated cell sorting assay

Cell sorting was performed on a Sony SH800 Cell Sorter (Sony Corporation, Japan). Briefly, the PBMCs were incubated with the following Ab-conjugates for 30 min on ice in the dark: anti-CD3-FITC (HIT3a, 300305, 1:100, BioLegend), anti-CD8-PC5.5 (SK1, 344709, 1:100, BioLegend), and anti-CD57-PB (HCD57, 322315, 1:100, BioLegend). Following incubation, the stained PBMCs were washed and resuspended in FACS buffer. Propidium iodide (PI; Life Technologies) was added to the cells immediately before analysis. CD57^+^CD8^+^ and CD57^-^CD8^+^ T cells were collected.

### RNA-seq

#### RNA isolation and library preparation

Total RNA was extracted with the QIAGEN RNeasy Micro Kit (Invitrogen, CA, USA). RNA purity and quantity were evaluated using a NanoDrop 2000 spectrophotometer (Thermo Fisher Scientific, USA). RNA integrity was measured with the Agilent 2100 Bioanalyzer (Agilent Technologies, Santa Clara, CA, USA). Libraries were constructed using the VAHTS Universal V6 RNA-seq Library Prep Kit. Transcriptome sequencing and analysis were performed by OE Biotech Co., Ltd. (Shanghai, China).

#### RNA sequencing and differentially expressed genes analysis

The libraries were sequenced using an Illumina NovaSeq 6000 platform, and 150 bp paired-end reads were generated. For each sample, approximately 50 M raw reads were generated. Raw reads in fastq format were processed using fastp^39^ and low-quality reads were removed. A total of 45 M clean reads for each sample were retained for further analysis. The clean reads were mapped to the reference genome using HISAT2 software^40^. Each gene’s FPKM^41^ was calculated, and its read counts were obtained using HTSeq-count^42^. Principal component analysis was performed to evaluate the biological duplication of the samples using the R (v 3.2.0).

Differential expression analysis was performed utilizing DESeq2^43^. Significant DEGs were defined as *Q* < 0.05, fold change >2, or fold change <0.5. Hierarchical cluster analysis of DEGs was performed using the R (v 3.2.0) to illustrate the expression patterns of genes among different groups and samples. A radar map of the top 30 genes was created using the R packet ggradar to illustrate the expression of the up- or down-regulated DEGs.

GO^44^ and KEGG pathway enrichment analyses^45^ of the DEGs were performed based on the hypergeometric distribution using the R (v 3.2.0) to identify significantly enriched terms. Column and bubble diagrams of the significant enrichment terms were drawn using the R (v 3.2.0).

GSEA was carried out with GSEA software^46,47^. Genes were ranked according to their degree of differential expression between the two groups, using a predefined gene set. We tested whether the predefined gene set was enriched at the top or bottom of the ranking list.

### Statistical analysis

All statistical analyses were performed using GraphPad Prism 9.5 (GraphPad Software, Inc., San Diego, CA, USA) and SPSS (version 22.0; SPSS Inc., Chicago, IL, USA). Comparisons were performed using two-tailed paired or unpaired Student’s *t*-test. The data in the boxplots are presented with the central mark indicating the median and the bottom and top edges of the box showing the 25th and 75th percentiles, respectively. The data in bar graphs are presented as the means ± SD. Group comparisons of categorical data were performed using Fisher’s exact test. The statistical significance threshold was set at *p* < 0.05.

### Written informed consent statement and ethical approval

The study was conducted in accordance with all relevant ethical regulations including the Declaration of Helsinki the Declaration of Helsinki. All patients, their legally acceptable representatives, or both (if possible) provided written informed consent. This study was approved by the relevant Institutional Review Board of the First Affiliated Hospital, College of Medicine, Zhejiang University (approval number: 2019-1371). In accordance with the ‘Guidance of the Ministry of Science and Technology (MOST) for the Review and Approval of Human Genetic Resources’ in China, formal approval for the export of human genetic material or data was obtained from the relevant authorities.

### Reporting summary

Further information on research design is available in the Nature Research Reporting Summary linked to this article.

### Supplementary information

Supplementary information

Reporting Summary

## Data Availability

Sequencing data were uploaded to the Sequence Read Archive (SRA) database of the National Center for Biotechnology Information (NCBI) (https://www.ncbi.nlm.nih.gov/sra/), with the BioProject ID accession number: PRJNA1045242. The patient data that support the findings of this study are available on request from the corresponding author (zhoujy@zju.edu.cn).

## Code availability

This study made use of publicly available packages with R version 3.2.0. The code that supports the findings of this study are available from the corresponding author on reasonable request.
