# Supplementary material for: CD57-positive CD8 + T cells define the response to anti-programmed cell death protein-1 immunotherapy in patients with advanced non-small cell lung cancer
Source: NPJ Precis Oncol. 2024 Jan 31;8:25. doi: 10.1038/s41698-024-00513-0 (PMC10830454; doi:10.1038/s41698-024-00513-0)
Supplement: Supplementary file 1 — Supplementary information [file 41698_2024_513_MOESM1_ESM.pdf]

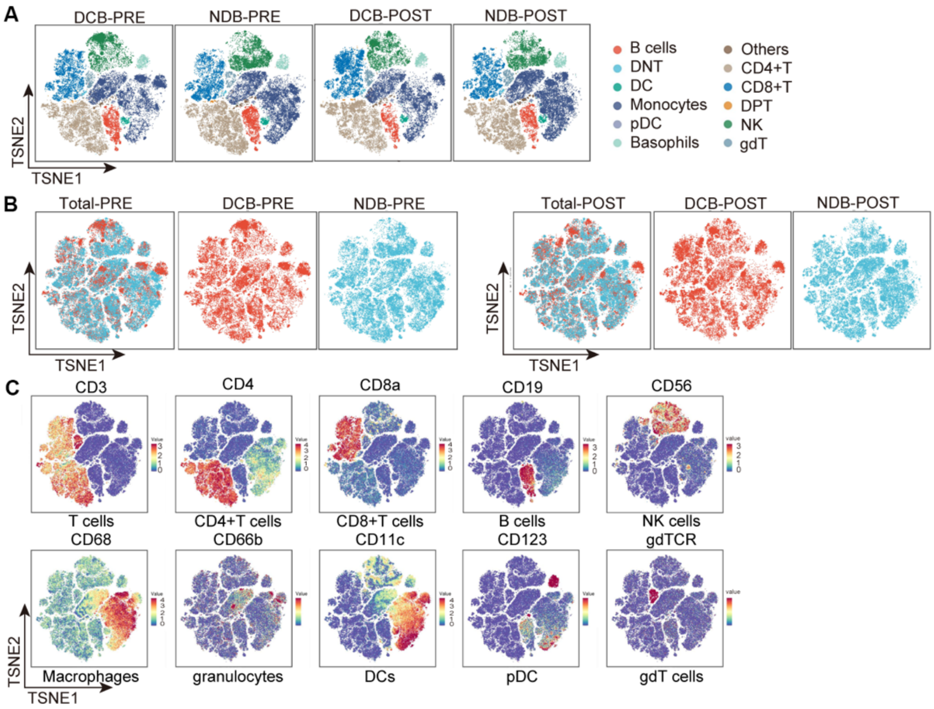

Supplementary Fig. 1 Major immune lineages of PBMCs from patients with NSCLC revealed by CyTOF.

a t-SNE plot identifying the 12 major immune cell subsets from PBMCs, including $CD4^+$ T cells, $CD8^+$ T cells, γδT, DNT, DPT, monocytes, DC, pDC, B cells, NK cells, basophils and other cells in DCB and NDB patients before and after immunotherapy, colored by major immune cell subsets. **b** t-SNE maps displaying 30,000 cells from the PBMC analyzed with immune cell subsets in DCB and NDB patients before and after immunotherapy, colored by patient groups. **c** t-SNE analysis defining the major immune cell subsets according to the expression of the main surface markers. PBMCs, peripheral blood mononuclear cells; NSCLC, non-small cell lung cancer; CyTOF, cytometry by time of flight; t-SNE, t-distributed Stochastic Neighbor Embedding; DNT, double-negative T cells; DPT, double-positive T cells; DC, dendritic cells; pDC, plasmacytoid dendritic cells; NK cells, natural killer cells; DCB, durable clinical benefit; NDB, no durable clinical benefit.

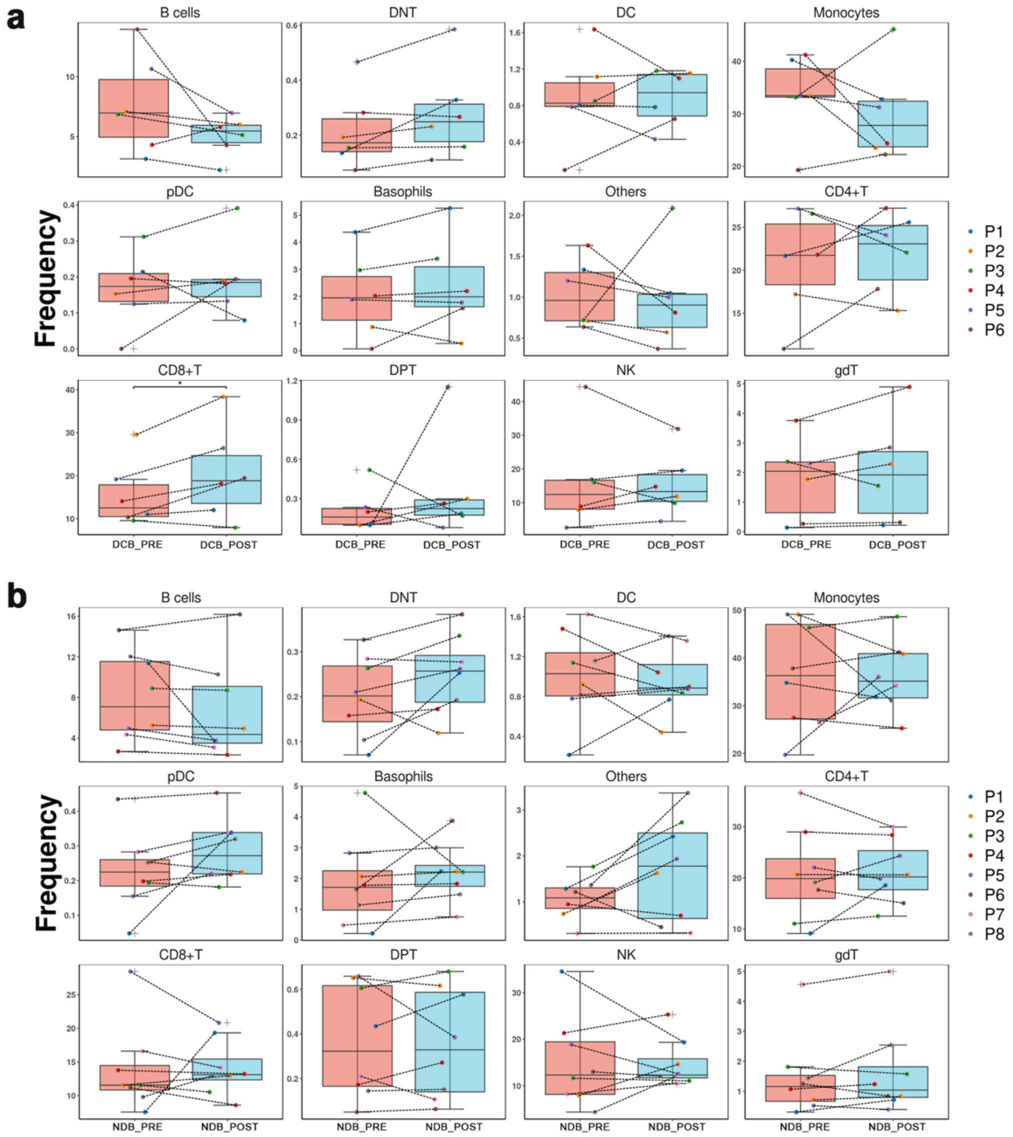

Supplementary Fig. 2 Paired analysis of PBMCs from patients with NSCLC revealed by CyTOF.

a, b Paired PBMC samples analysis before and after immunotherapy demonstrating the changes in frequencies of the 12 immune cell subsets among (**a**) DCB and (**b**) NDB patients. *$p < 0.05$.

PBMCs, peripheral blood mononuclear cells; NSCLC, non-small cell lung cancer; CyTOF, cytometry by time of flight; DCB, durable clinical benefit; NDB, no durable clinical benefit.

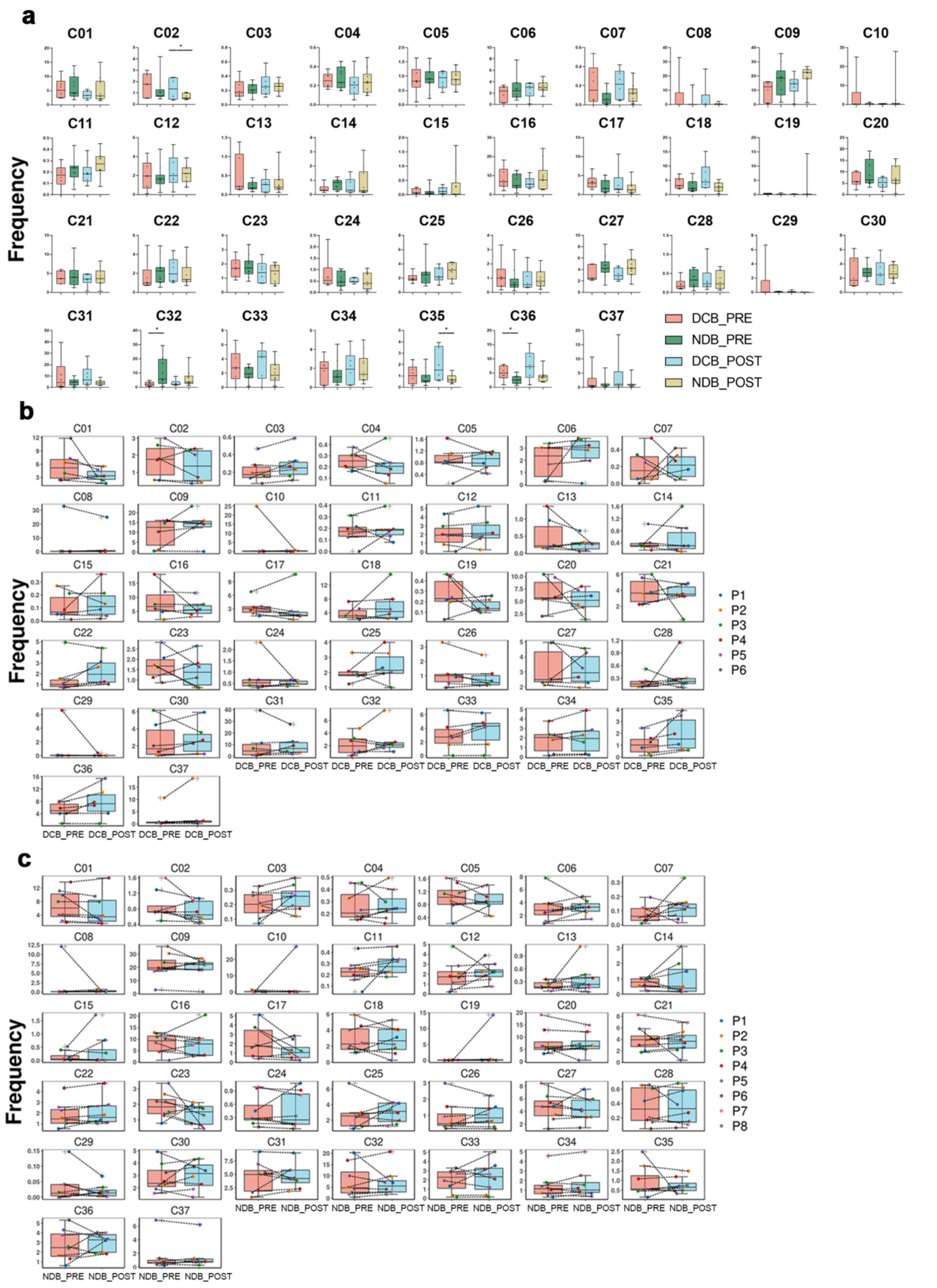

Supplementary Fig. 3 Analysis of the peripheral immune cell populations in patients with NSCLC.

a Boxplots demonstrating the frequencies of the 37 immune cell clusters among DCB and NDB patients before and after immunotherapy. **b** Paired PBMC samples analysis

before and after immunotherapy demonstrating the changes in frequencies of the 37 immune cell clusters among DCB and (**c**) NDB patients. *$p < 0.05$.

NSCLC, non-small cell lung cancer; DCB, durable clinical benefit; NDB, no durable clinical benefit; PBMC, peripheral blood mononuclear cell.

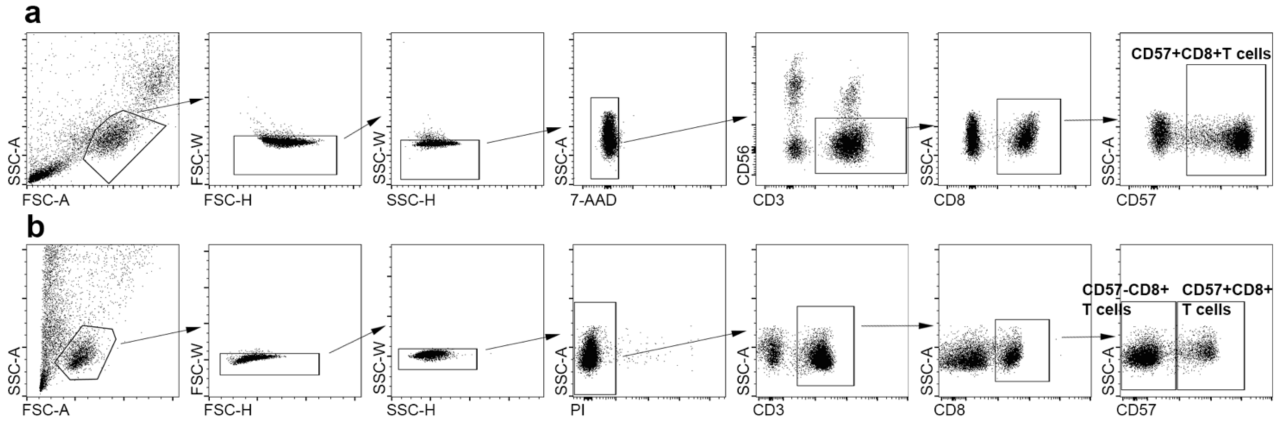

Supplementary Fig. 4 All FACS sequential gating/sorting strategies.

a Gating strategy to determine the percentage of $CD57^{+}CD8^{+}$ T cells. **b** Gating strategy to sort $CD57^{+}CD8^{+}$ T cells and $CD57^{-}CD8^{+}$ T cells from patients with NSCLC treated with PD-1 inhibitors.

NSCLC, non-small cell lung cancer; PD-1, programmed cell death protein 1.

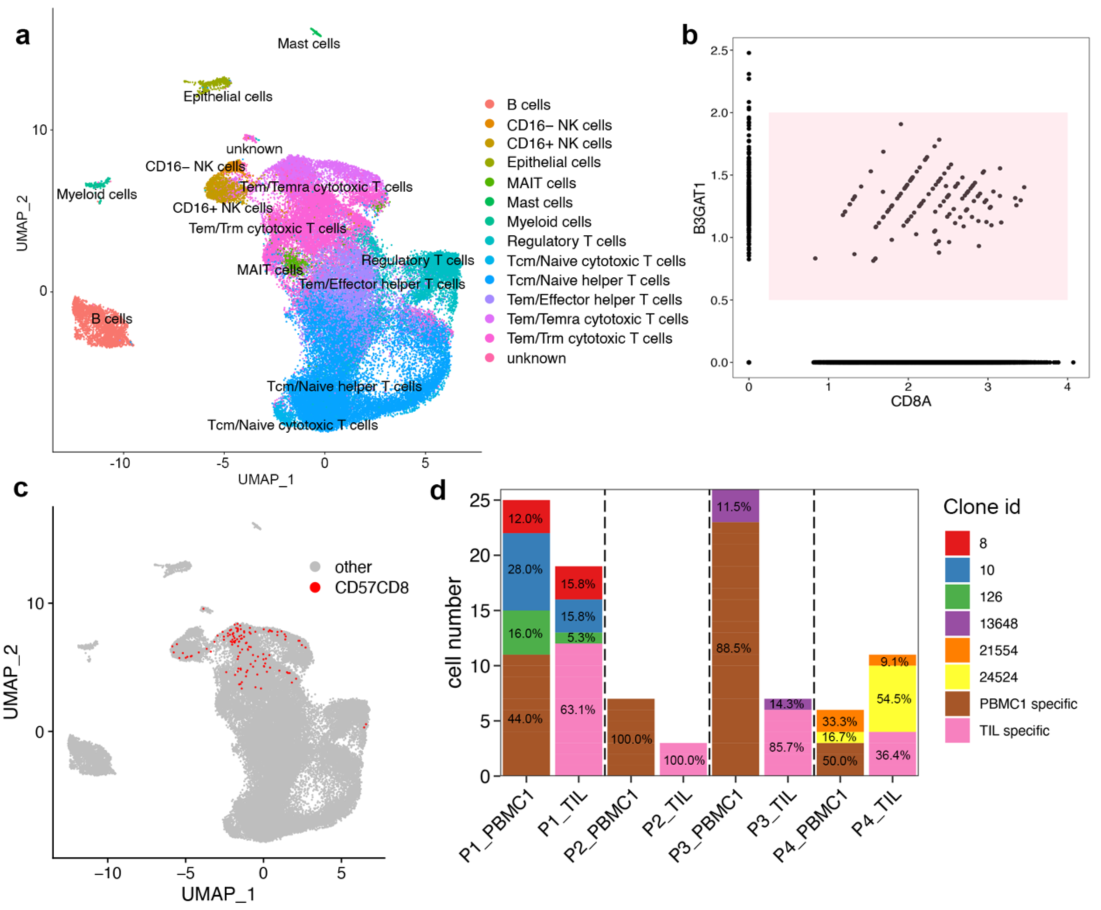

Supplementary Fig.5 TCR Clonality of CD57$^+$CD8$^+$ T Cells in Blood and Tissue.

a UMAP plot of all cells colored by cell types. **b** Selection of CD57$^+$CD8$^+$ T cells based on the expression of B3GAT1 (CD57) and CD8A. **c** In the UMAP plot, CD57$^+$CD8$^+$ T cells are represented by red color, distinguishing them from other cell populations. **d** Among the 4 patients, except for patient 2, the presence of the same TCR clones in CD57$^+$CD8$^+$ T cells is observed in both the blood and tissue samples. The clone id numbers represent unique TCR clone IDs that are present in both the blood and tissue compartments, indicating shared TCR clones between the two compartments. 'PBMC specific' indicates TCR clones found exclusively in the blood samples, while 'TIL specific' indicates TCR clones found exclusively in the tissue samples.

TCR, T-cell receptor; UMAP, Uniform Manifold Approximation and Projection; PBMC, peripheral blood mononuclear cell; TIL, tumor-infiltrating lymphocytes.

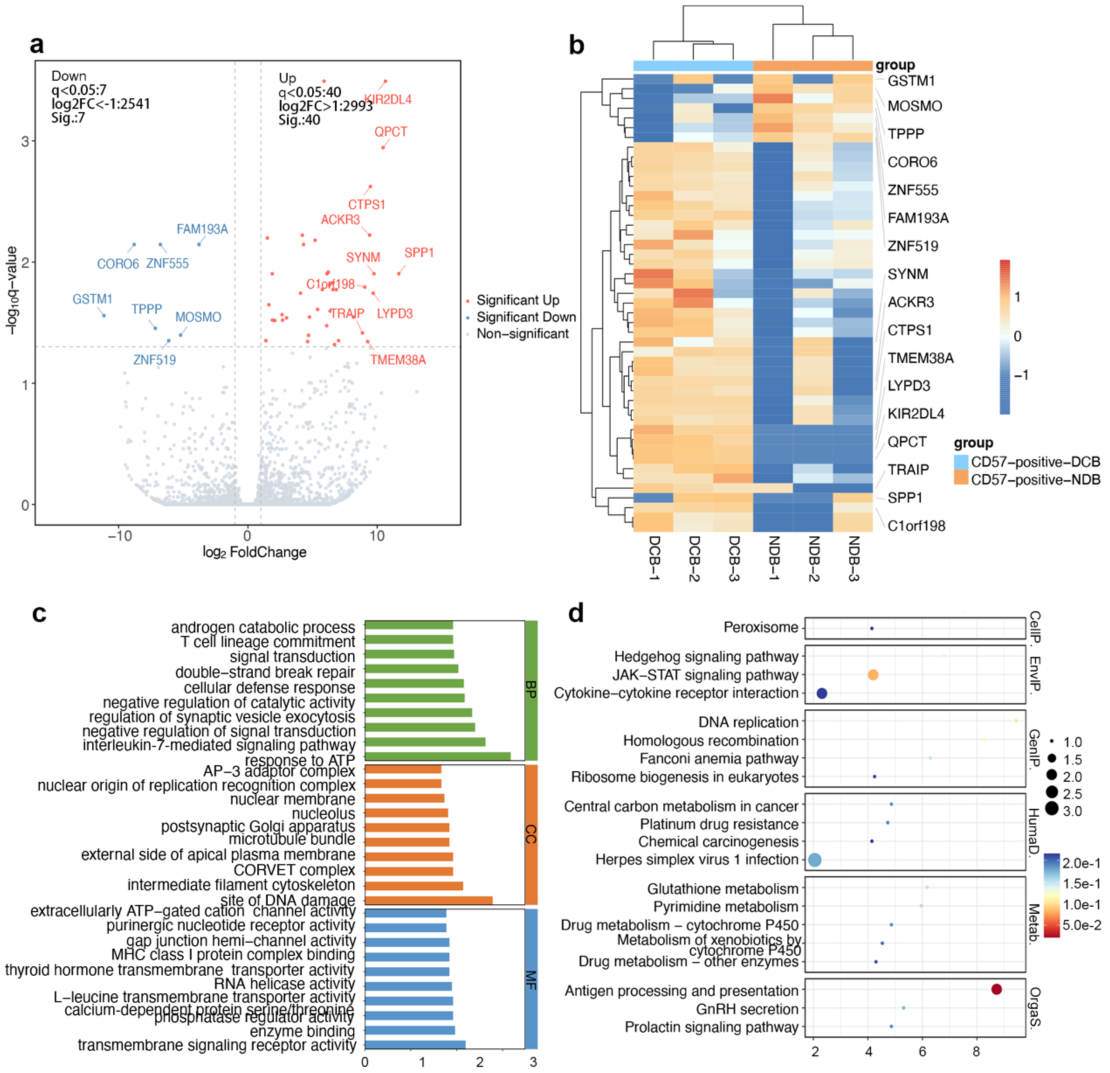

Supplementary Fig. 6 Identification of DEGs and screening of genes-based GO and KEGG analysis in CD57$^+$CD8$^+$ T cells between DCB and NDB patients.

a Volcano plot showing the 47 DEGs between DCB patients and NDB patients, including 40 upregulated genes and 7 downregulated genes. Red and blue colors represent upregulated and downregulated genes, respectively. **b** Clustering analysis of DEGs and samples. The color scale bar for heat intensity indicates Log2(Fold Change). Columns, samples; rows, DEGs. The samples were grouped into two distinct clusters: DCB cluster and NDB cluster. **c** GO analysis of DEGs. The most enriched 10 GO terms in biological process, cellular component, and molecular function, separately. The y axis represents GO terms and the x axis represents the value of -log10 (*p*-value). **d** KEGG enrichment analysis of DEGs. The x axis represents enrichment score and the y axis represents pathway. Size and color of the

bubble represent the amount of DEGs enriched in pathway and enrichment significance, respectively.

DEGs, differentially expressed genes; GO, Gene Ontology; KEGG, Kyoto Encyclopedia of Genes and Genomes; GSEA, Gene Set Enrichment Analysis; DCB, durable clinical benefit; NDB, no durable clinical benefit.

Supplementary Table 1. CyTOF panel. Antibodies were obtained from Fluidigm (South San Francisco, CA, USA).

List	Label	marker
1	89Y	CD57
2	115ln	CD3
3	139La	CD68
4	141Pr	CD56
5	142Nd	gdTCR
6	143Nd	CCR6
7	144Nd	CD14
8	145Nd	IgD
9	146Nd	CD123
10	147Sm	CD85j
11	148Nd	CD19
12	149Sm	CD25
13	150Nd	PDL1
14	151Eu	ICOS
15	152Sm	CD39
16	153Eu	CD27
17	154Sm	CD24
18	155Gd	CD45RA
19	156Gd	CD86
20	157Gd	CD28
21	158Gd	CCR7
22	159Tb	CD11c
23	160Gd	CD33
24	161Dy	CTLA_4
25	162Dy	FoxP3
26	163Dy	CD161
27	164Dy	CXCR5
28	165Ho	CD66b
29	166Er	CXCR3
30	167Er	CD94
31	168Er	Tbet
32	169Tm	Ki67
33	170Er	CD127
34	171Yb	PD1
35	172Yb	CD38
36	173Yb	Granzyme B
37	174Yb	CD20
38	175Lu	CD16
39	176Yb	HLA_DR
40	197gd	CD4

List	Label	marker
41	198pt	CD8a
42	209Bi	CD11b

CyTOF, cytometry by time of flight.

Supplementary Table 2. Blood-collection from for the NSCLC patient

Patient number	Group	Prior to therapy	12 weeks after therapy
Patient 1	DCB	Yes	Yes
Patient 2	DCB	Yes	Yes
Patient 3	DCB	Yes	Yes
Patient 4	DCB	Yes	Yes
Patient 5	DCB	Yes	Yes
Patient 6	DCB	Yes	Yes
Patient 7	NDB	Yes	Yes
Patient 8	NDB	NA	Yes
Patient 9	NDB	Yes	Yes
Patient 10	NDB	Yes	Yes
Patient 11	NDB	Yes	Yes
Patient 12	NDB	Yes	NA
Patient 13	NDB	Yes	Yes
Patient 14	NDB	Yes	Yes
Patient 15	NDB	Yes	Yes
Patient 16	NDB	Yes	Yes
Patient 17	NDB	NA	Yes
Patient 18	NDB	Yes	NA
Patient 19	NDB	Yes	NA
Patient 20	Death	Yes	NA

"Yes" represented collected blood samples, while "NA" represented blood samples were not collected.

Supplementary Table 3. Comparison between patients with high and low $CD57^{+}CD8^{+}$ T cells/T cells ratio.

**Characteristics**	**$CD57^{+}CD8^{+}$T cells/T cells<12.85% (n=13)**	**$CD57^{+}CD8^{+}$T cells/T cells≥12.85% (n=14)**	***P* valvue**
Gender			0.496
Male (M)	11(84.6%)	13(92.9%)	
Female (F)	2(15.4%)	1(7.1%)	
Age (years)			0.901
<65	4(30.8%)	4(28.6%)	
≥65	9(69.2%)	10(71.4%)	
Smoking			0.557
YES	3(23.1%)	2(14.3%)	
NO	10(76.9%)	12(85.7%)	
Histological type			0.842
LUAD	7(53.8%)	7(50.0%)	
LUSC	6(46.2%)	7(50.0%)	
Tumor invasion (T_stage)			0.148
≤3	3(23.1%)	7(50.0%)	
4	10(76.9%)	7(50.0%)	
Lymph node (N_stage)			0.332
≤2	8(61.5%)	6(42.9%)	
3	5(38.5%)	8(57.1%)	
Metastasis (M_stage)			0.686
M0	2(15.4%)	3(21.4%)	
M1	11(84.6%)	11(78.6%)	
Tumor stage			0.686
Stage IIIB/C	2(15.4%)	3(21.4%)	
Stage IV	11(84.6%)	11(78.6%)	

LUAD, lung adenocarcinoma; LUSC, lung aquamous cell carcinoma.

Supplementary Table 4. The clinical characteristics of advanced NSCLC in FFPE cohort

**Characteristics**	**Validation cohort (n=90)**	**DCB (n=44)**	**NDB (n=46)**	***p* valvue**
Gender				0.205
Male (M)	80(88.9%)	41(93.2%)	39(84.8%)	
Female (F)	10(11.1%)	3(6.8%)	7(15.2%)	
Age (years)				0.075
<65	35(38.9%)	13(29.5%)	22(47.8%)	
≥65	55(61.1%)	31(70.5%)	24(52.2%)	
Smoking				0.905
YES	68(75.6%)	33(75.0%)	35(76.1%)	
NO	22(24.4%)	11(25.0%)	11(23.9%)	
Line of therapy				0.815
First	50(55.6%)	28(63.6%)	22(47.8%)	
Second	40(44.4%)	16(36.4%)	24(52.2%)	
Histological type				0.084
LUAD	53(58.9%)	31(70.5%)	22(47.8%)	
LUSC	35(38.9%)	12(27.3%)	23(50.0%)	
Others	2(2.2%)	1(2.3%)	1(2.2%)	
Tumor stage				0.260
Stage IIIB/C	20(22.2%)	12(27.3%)	8(17.4%)	
Stage IV	70(77.8%)	32(72.7%)	38(82.6%)	

NSCLC, non-small cell lung cancer; FFPE, Formalin-Fixed Paraffin-Embedded; DCB, durable clinical benefit; NDB, no durable clinical benefit; LUAD, lung adenocarcinoma; LUSC, lung squamous cell carcinoma.
