# Supplementary material for: CD57-positive CD8 + T cells define the response to anti-programmed cell death protein-1 immunotherapy in patients with advanced non-small cell lung cancer
Source: NPJ Precis Oncol. 2024 Jan 31;8:25. doi: 10.1038/s41698-024-00513-0 (PMC10830454; doi:10.1038/s41698-024-00513-0)
Supplement: Supplementary file 2 — Reporting Summary [file 41698_2024_513_MOESM2_ESM.pdf]

nature portfolio

Corresponding author(s): Jianya Zhou, Xun Zeng

Last updated by author(s): Dec 1, 2023

Reporting Summary

Nature Portfolio wishes to improve the reproducibility of the work that we publish. This form provides structure for consistency and transparency in reporting. For further information on Nature Portfolio policies, see our Editorial Policies and the Editorial Policy Checklist.

Statistics

For all statistical analyses, confirm that the following items are present in the figure legend, table legend, main text, or Methods section.

n/a	Confirmed	
☐	☒	The exact sample size (*n*) for each experimental group/condition, given as a discrete number and unit of measurement
☐	☒	A statement on whether measurements were taken from distinct samples or whether the same sample was measured repeatedly
☐	☒	The statistical test(s) used AND whether they are one- or two-sided<br>*Only common tests should be described solely by name; describe more complex techniques in the Methods section.*
☐	☒	A description of all covariates tested
☐	☒	A description of any assumptions or corrections, such as tests of normality and adjustment for multiple comparisons
☐	☒	A full description of the statistical parameters including central tendency (e.g. means) or other basic estimates (e.g. regression coefficient) AND variation (e.g. standard deviation) or associated estimates of uncertainty (e.g. confidence intervals)
☐	☒	For null hypothesis testing, the test statistic (e.g. F, t, r) with confidence intervals, effect sizes, degrees of freedom and P value noted<br>*Give P values as exact values whenever suitable.*
☐	☒	For Bayesian analysis, information on the choice of priors and Markov chain Monte Carlo settings
☐	☒	For hierarchical and complex designs, identification of the appropriate level for tests and full reporting of outcomes
☐	☒	Estimates of effect sizes (e.g. Cohen's *d*, Pearson's *r*), indicating how they were calculated

Our web collection on statistics for biologists contains articles on many of the points above.

Software and code

Policy information about availability of computer code

Data collection	No software and code were used.
Data analysis	Fastp is open-source code and corresponding instructions are available at https://github.com/OpenGene/fastp.<br>HISAT is open-source software freely available at http://www.ccb.jhu.edu/software/hisat/.<br>HTSeq is released as an open-source software under the GNU General Public Licence and available from http://www-huber.embl.de/HTSeq or from the Python Package Index at https://pypi.python.org/pypi/HTSeq.<br>The DESeq2 package is available at http://www.bioconductor.org/packages/release/bioc/html/DESeq2.html webcite.<br>The Gene Ontology resource is available at GO; http://geneontology.org).<br>KEGG is available at http://www.genome.jp/kegg/.<br>Data generated from different batches were normalized through bead normalization method. Software implementing this method is freely available for download from www.cytobank.org/nolanlab.<br>Source code for PhenoGraph is available online for MATLAB and Python (www.c2b2.columbia.edu/danapeerlab/html/software.html).

For manuscripts utilizing custom algorithms or software that are central to the research but not yet described in published literature, software must be made available to editors and reviewers. We strongly encourage code deposition in a community repository (e.g. GitHub). See the Nature Portfolio guidelines for submitting code & software for further information.

Data

Policy information about availability of data

All manuscripts must include a data availability statement. This statement should provide the following information, where applicable:

- Accession codes, unique identifiers, or web links for publicly available datasets
- A description of any restrictions on data availability
- For clinical datasets or third party data, please ensure that the statement adheres to our policy

Sequencing data were uploaded to the Sequence Read Archive (SRA) database of the National Center for Biotechnology Information (NCBI) (https://www.ncbi.nlm.nih.gov/sra/), with the BioProject ID accession number: PRJNA1045242. The patient data that support the findings of this study are available on request from the corresponding author (zhoujy@zju.edu.cn).

Research involving human participants, their data, or biological material

Policy information about studies with human participants or human data. See also policy information about sex, gender (identity/presentation), and sexual orientation and race, ethnicity and racism.

Reporting on sex and gender	We analyzed gender comparisons between groups of patients to determine whether there were differences between the sexes.
Reporting on race, ethnicity, or other socially relevant groupings	we did not used the socially constructed or socially relevant categorization variable's.
Population characteristics	We used covariate-relevant population characteristics of the human research participants, including age, past and current diagnosis and treatment categories.
Recruitment	The study included patients with advanced NSCLC who were treated with PD-1 inhibitors at The First Affiliated Hospital, College of Medicine, Zhejiang University (China). Patients diagnosed with clinical stage IIIB/IIIC enrolled in this study were deemed inoperable by the lung multidisciplinary team and suitable for immunotherapy. Pathological or clinical staging was performed according to the eighth edition of the American Joint Committee on Cancer guidelines.
Ethics oversight	This study was approved by the relevant Institutional Review Board of the First Affiliated Hospital, College of Medicine, Zhejiang University (approval number: 2019-1371). Patients provided written informed consent to participate in these studies.

Note that full information on the approval of the study protocol must also be provided in the manuscript.

Field-specific reporting

Please select the one below that is the best fit for your research. If you are not sure, read the appropriate sections before making your selection.

☒ Life sciences ☐ Behavioural & social sciences ☐ Ecological, evolutionary & environmental sciences

For a reference copy of the document with all sections, see nature.com/documents/nr-reporting-summary-flat.pdf

Life sciences study design

All studies must disclose on these points even when the disclosure is negative.

Sample size	We collected all available samples during the collection period.
Data exclusions	Chinese patients with advanced-stage NSCLC treated with PD-1 inhibitor at the First Affiliated Hospital, College of Medicine, Zhejiang University, were included in this study.
Replication	Flow cytometry analysis was performed in two separate cohorts. Cohort 1 consisted of 27 NSCLC patients treated with single-agent PD-1 inhibitors between May 2021 and April 2022. Cohort 2 included 48 NSCLC patients treated with a combination of immunotherapy and platinum-based chemotherapy between May 2021 and December 2022.
Randomization	Treatment response was investigator-assessed based on the Response Evaluation Criteria in Solid Tumors version 1.1. Response to immunotherapy was classified into a DCB (complete response, partial response, or stable disease (SD) lasting >6 months) and NDB (progressive disease or SD lasting <6 months).
Blinding	Investigators were blinded to group allocation.

Reporting for specific materials, systems and methods

We require information from authors about some types of materials, experimental systems and methods used in many studies. Here, indicate whether each material, system or method listed is relevant to your study. If you are not sure if a list item applies to your research, read the appropriate section before selecting a response.

Materials & experimental systems

n/a	Involved in the study
☐	☒ Antibodies
☒	☐ Eukaryotic cell lines
☒	☐ Palaeontology and archaeology
☒	☐ Animals and other organisms
☒	☐ Clinical data
☒	☐ Dual use research of concern
☒	☐ Plants

Methods

n/a	Involved in the study
☒	☐ ChIP-seq
☐	☒ Flow cytometry
☒	☐ MRI-based neuroimaging

Antibodies

Antibodies used

Anti-CD66b-PE-Cy7 (G10F5, BioLegend, 305115), anti-CD3-BV510 (UCHT1, BioLegend, 300447), anti-CD56-BV711 (HCD56, BioLegend, 318335), anti-CD8a-APC-Cy7 (RPA-T8, BioLegend, 301015), anti-CD57-PB (HCD57, BioLegend, 322315), 7-AAD (BioLegend, 420404), CD3 (D7A6E, CST, 85061), CD57 (HNK-1, CST, 72031)), CD8 (EPR22483-288, Abcam, ab245118), anti-CD3-FITC (HIT3a, BioLegend, 300305), anti-CD8-PC5.5 (SK1, BioLegend, 344709), and anti-CD57-PB (HCD57, BioLegend, 322315)

Validation

Anti-CD66b-PE-Cy7: Verified Reactivity in Human, application in flow. Anti-CD3-BV510: Verified Reactivity in Human, application in flow. Anti-CD56-BV711: Verified Reactivity in Human, application in flow. Anti-CD8a-APC-Cy7: Verified Reactivity in Human, application in flow. Anti-CD57-PB: Verified Reactivity in Human, application in flow. 7-AAD: Application FC-Quality tested. CD3 suitable for IHC-P, WB, IP. Reacts with: Human, monkey. CD57 Suitable for:IHC-P, IF. Reacts with: Human. CD8: Suitable for: IHC-P, Flow Cyt, IP Reacts with: Human. Anti-CD3-FITC: Verified Reactivity in Human, application in flow. Anti-CD8-PC5.5: Verified Reactivity in Human, application in flow. Anti-CD57-PB (HCD57, BioLegend, 322315): Verified Reactivity in Human, application in flow.

Plants

Seed stocks

not applicable

Novel plant genotypes

not applicable

Authentication

not applicable

Flow Cytometry

Plots

Confirm that:

☒ The axis labels state the marker and fluorochrome used (e.g. CD4-FITC).

☒ The axis scales are clearly visible. Include numbers along axes only for bottom left plot of group (a 'group' is an analysis of identical markers).

☒ All plots are contour plots with outliers or pseudocolor plots.

☒ A numerical value for number of cells or percentage (with statistics) is provided.

Methodology

Sample preparation

For PBMC isolation, we collected 10ml fresh whole blood from patients with NSCLC in K2EDTA-coated vacutainer tubes (BD Biosciences). Ficoll-Paque PLUS (GE Healthcare) was used to separate PBMCs by density gradient centrifugation. Subsequently, washes were performed with the FACS buffer (PBS+0.5% bovine serum albumin) twice at 400 g for 10 min at room temperature. PBMCs were resuspended in the FACS buffer and counted.PBMCs were incubated with the following Ab-conjugates on ice in the dark: anti-CD66b-PE-Cy7 (G10F5), anti-CD3-BV510 (UCHT1), anti-CD56-BV711 (HCD56), anti-CD8a-APC-Cy7 (RPA-T8), anti-CD57-PB (HCD57). Incubation was completed for 30 minutes, then samples were washed twice with

	FACS buffer and resuspended in 300 μL of the same buffer. Furthermore, 7-AAD (BioLegend) was added to PBMCs immediately before analysis. The flow cytometry was carried out using a BD FACSFortessa Multicolor Flow Cytometer (BD Biosciences).
Instrument	BD FACSDiva Software v8.0.1
Software	Flowjo
Cell population abundance	The CD57+CD8+T cell/T cell ratio was calculated using the following formula: (CD57+CD8+CD3+cells)/(CD3+cells) 100%. The CD57+CD8+T cells/CD8+T cells ratio was calculated using the formula below: (CD57+CD8+CD3+cells)/(CD8+CD3+cells) 100%. As well, the total CD8+T cell/T cell ratio and the total CD57+T cell/T cell ratio were calculated as the proportion of CD8+CD3+cells among total CD3+cells and the proportion of CD57+CD3+cells among total CD3+cells, respectively.
Gating strategy	FSC/SSC gates of the starting lymphocyte population FSC-A/FSC-H and SSC-A/SSC-H were used to remove desadhesions, 7AAD-negative to sort live cells, CD3+ to gate T cells, finally gating CD8+CD57+.

☒ Tick this box to confirm that a figure exemplifying the gating strategy is provided in the Supplementary Information.
